# Production of Methyl Lactate with Sn-USY and Sn-β: Insights into Real Hemicellulose Valorization

**DOI:** 10.1021/acssuschemeng.3c07356

**Published:** 2024-02-06

**Authors:** Jose M. Jiménez-Martin, Miriam El Tawil-Lucas, Maia Montaña, María Linares, Amin Osatiashtiani, Francisco Vila, David Martín Alonso, Jovita Moreno, Alicia García, Jose Iglesias

**Affiliations:** † Chemical & Environmental Engineering Group, Universidad Rey Juan Carlos, C/Tulipan s/n, 28933 Madrid, Spain; ‡ Energy & Bioproducts Research Institute (EBRI), College of Engineering and Physical Sciences, Aston University, Aston Triangle, Birmingham B4 7ET, United Kingdom; § Energy and Sustainable Chemistry (EQS) Group, Institute of Catalysis and Petrochemistry, CSIC, C/Marie Curie 2, Campus de Cantoblanco, 28049 Madrid, Spain; ∥ Instituto de Tecnologías para la Sostenibilidad. Universidad Rey Juan Carlos. C/Tulipan s/n, 28933. Madrid, Spain

**Keywords:** zeolites, Sn-USY, Sn-β, biomass, hemicellulose, retroaldol reaction, methyl lactate

## Abstract

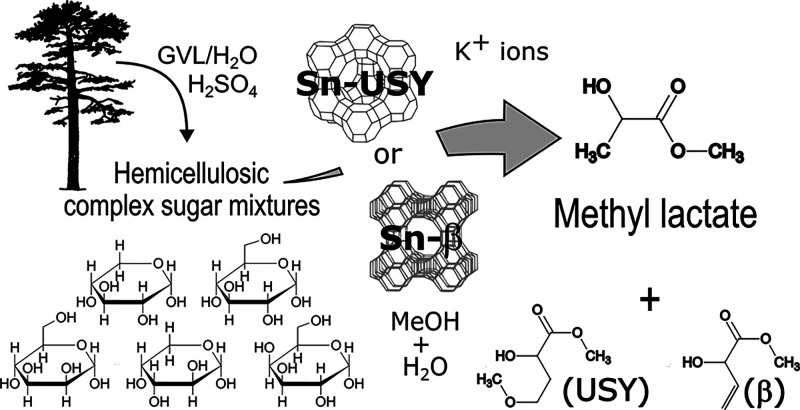

Potassium exchanged Sn-β and Sn-USY zeolites have been tested for the transformation of various aldoses (hexoses and pentoses), exhibiting outstanding catalytic activity and selectivity toward methyl lactate. Insights into the transformation pathways using reaction intermediates—dihydroxyacetone and glycolaldehyde—as substrates revealed a very high catalytic proficiency of both zeolites in aldol and retro-aldol reactions, showcasing their ability to convert small sugars into large sugars, and vice versa. This feature makes the studied Sn-zeolites outstanding catalysts for the transformation of a wide variety of sugars into a limited range of commercially valuable alkyl lactates and derivatives. [K]Sn-β proved to be superior to [K]Sn-USY in terms of shape selectivity, exerting tight control on the distribution of produced α-hydroxy methyl esters. This shape selectivity was evident in the transformation of several complex sugar mixtures emulating different hemicelluloses—sugar cane bagasse, Scots pine, and white birch—that, despite showing very different sugar compositions, were almost exclusively converted into methyl lactate and methyl vinyl glycolate in very similar proportions. Moreover, the conversion of a real hemicellulose hydrolysate obtained from Scots pine through a simple GVL-based organosolv process confirmed the high activity and selectivity of [K]Sn-β in the studied transformation, opening new pathways for the chemical valorization of this plentiful, but underutilized, sugar feedstock.

## Abstract 2

Sn-β zeolite shows outstanding catalytic activity in the transformation of complex sugar mixtures (hemicelluloses) to methyl lactate through aldol and retro-aldol condensation pathways.

## Introduction

Alkyl lactates are small, highly functionalized, biomass-derived molecules. They are interesting not only because of the numerous applications they find—such as being a monomer for poly(lactic acid) (PLA) and highly biodegradable green solvents—but also as a starting point in the synthesis of a wide variety of chemicals,^1^ including acrylic acid, 1,2-propanediol, pyruvic acid, acetaldehyde, and many other products, being considered important chemical building blocks.^2^

The synthesis of alkyl lactates can be tackled through different approaches,^3^ namely, esterification of lactic acid,^4^ alcoholytic depolymerization of PLA,^5^ dehydrogenation and isomerization of glycerol,^6^ or the retro-aldol reaction of monosaccharides.^7^ This last has garnered the attention from the scientific community in the past decade given the substantial number of published works on this topic.^7−9^ Most notable reported catalysts to drive this transformation are those based on tin-functionalized zeolites.^10,11^ Sn-β zeolite is the most studied catalytic system,^12−15^ although some other zeotype materials have proven also to be highly active catalytic systems, providing very high yields toward alkyl lactates.^16−20^ The reaction mechanism has been described to proceed, in the presence of short alkyl chain alcohols, through the isomerization of the starting aldose to the corresponding ketose (fructose), which undergoes retro-aldol cleavage into two three-carbon backbone compounds, which evolve through a sequence of dehydration–acetalization–isomerization transformations to the target alkyl lactate.^21,22^ According to this mechanism, hexoses provide two molecules of lactate, whereas pentoses yield a single lactate molecule accompanied by a two-carbon moiety, glycolaldehyde, as depicted in Scheme S1.^23^ In this way, hexose-rich carbohydrates (e.g., cellulose, starch hydrolysates, and sucrose) are preferred because a higher productivity toward alkyl lactates is expected. This makes C5-sugar rich feedstock a much less attractive option for lactate production despite its comparatively higher abundance and easy production through chemical hydrolysis. Nevertheless, the reversibility of the retro-aldol reaction of carbohydrates in the presence of tin-functionalized materials, as demonstrated by Sádaba and co-workers,^24^ allows building C4 and C6 sugars from C2 fragments. This transformation, if adequately combined with retro-aldol cleavage of C5-sugars, opens an opportunity for the use of an underutilized pentose-rich feedstock to produce alkyl lactates. Despite this, only few works have been reported dealing with the conversion of pentose sugars into alkyl lactates, and most of them only tackle the transformation of xylose, the most abundant pentose sugar.^23,25−28^ Thus, the potential of pentoses as substrates for the production of valuable methyl lactate remains unexplored.

Within this work, we aim to take a further step in the simultaneous valorization of hexoses and pentoses and present the use of tin-functionalized zeolites in the transformation of complex sugar mixtures emulating hemicellulose from different feedstocks as well as real Scots pine hemicellulose into methyl lactate. These zeolites offer high catalytic activity in retro-aldol reactions and self-condensation of large and small sugars, thus promoting the formation of methyl lactate and related α-hydroxyesters in very high yields from complex sugar mixtures.

## Experimental Section

### Synthesis of Sn-Containing Zeolites

[K]Sn-USY and [K]Sn-β were prepared according to a postsynthetic metalation procedure previously reported.^29^ Typically, parent zeolites (USY: JCPDS card number 12-0246; β: JCPDS card number 48-0038; with starting SiO_2_/Al_2_O_3_ of 12 and 19, respectively) in NH_4_^+^ form were calcined at 550 °C for 6 h and contacted with concentrated nitric acid aqueous solutions (10 mol·L^–1^) for 1 h under stirring at room temperature to drive the dealumination of the zeolites, which creates framework vacancies. The dealumination procedure was repeated twice to ensure maximum removal of aluminum. Metalation was achieved by contacting zeolite suspensions in CH_2_Cl_2_ with tin chloride for 5 h at room temperature to allow the diffusion of the tin precursor inside the porous structure. An appropriate amount of SnCl_4_·5H_2_O, enough to achieve 2 wt % Sn loding in the zeolite, assuming quantitative incorporation, was dissolved in a mixture of acetone/dichloromethane and treated with sodium sulfate to remove water. The filtered solution was added to the grafting media and allowed to diffuse in the porous structure of the considered zeolite for 5 h. The resultant mixtures were then treated with triethylamine (molar ratio SnCl_4_/NEt_3_ = 1:4) to promote the chemical grafting of SnCl_4_ in aluminum vacancies. The catalysts were then recovered by centrifugation, air-dried, and calcined at 550 °C for 6 h. The Bro̷nsted acidity of the Sn-functionalized zeolites was neutralized by ion exchange with KCl.^29^

### Catalyst Characterization

The metal content in zeolites was evaluated through ICP-OES using a Varian Vista AX unit, previously calibrated using standard stock solutions. Textural properties were evaluated through argon manometric porosimetry recording adsorption–desorption isotherms at −186 °C using a Micromeritics Triflex unit. Surface area values were calculated using the B.E.T. equation, and the total pore volume was assumed to be that recorded at *P*/*P*_0_ = 0.95. X-ray powder diffraction (XRD) patterns were collected in a Philips X-pert diffractometer using the Cu Kα line in the 2θ angle range from 5 to 90° (step size of 0.04°). Acidity was measured by temperature-programmed desorption of NH_3_ in a Micromeritics 2910 (TPD/TPR) equipment fitted with a TCD detector. Diffuse reflectance infrared Fourier transform (DRIFT) spectra of pyridine-adsorbed catalysts were obtained using a Nicolet 6700 (ThermoFisher Scientific, UK) with an infrared source and mercury cadmium telluride (MCT/A) photon detector at −196 °C, collecting 64 scans with 4 cm^–1^ resolution. Prior to pyridine adsorption, the catalysts were outgassed at 300 °C under a vacuum. Subsequently, the catalysts were wetted with pyridine and allowed to adsorb for 5 min, after which excess physisorbed pyridine was removed in a vacuum oven at 150 °C operating overnight. In the next step, 50 mg of each catalyst was diluted with 450 mg of finely ground KBr (spectroscopy grade, Fisher Chemical) to obtain a 10 wt % sample. The catalyst and KBr were thoroughly mixed to ensure homogeneity and composition consistency across all solid mixtures. The DRIFT spectra of the samples were collected using a Praying Mantis High Temperature Reaction Chamber (Harrick Scientific, USA) integrated into a Praying Mantis Diffuse Reflection Accessory. The powder samples (50 mg) were loaded into the sample cup of the reaction chamber, where the sample’s temperature was monitored by employing a thermocouple inserted directly into the sample holder, coming into direct contact with the powder. This allowed for additional drying under a vacuum at 150 °C for 10 min to eliminate any remaining physisorbed moisture prior to measurements. DR-UV–vis spectra were recorded at room temperature in a Varian Cary 500 unit fitted with a Praying Mantis accessory and an environmental cell (HVC-DRP Harrick Scientific Products, NY). Samples were conditioned prior to analysis by heating at 200 °C under a N_2_ flow (70 mL·g^–1^) for 1 h. Analysis of physisorbed species in spent catalysts was conducted by ^1^H and ^13^C NMR by contacting spent catalyst samples with CDCl_3_ (0.5 g catalyst/1 mL CDCl_3_) in an ultrasound bath for 30 min.

### Catalytic Tests

Catalytic tests were performed in a 100 mL capacity stainless steel batch reactor (Autoclave Engineers) fitted with a temperature and stirring controller and a pressure transducer to monitor the reaction conditions. Typically, a proper amount of substrate was dissolved in methanol (75 mL) together with the corresponding catalyst (1 g), *n*-decane (internal standard, 0.01 g·L^–1^), and, if applicable, water, altogether suspended in the reaction vessel. The reactor was sealed, the temperature was set at 150 °C, and the stirring rate was set at 500 rpm to conduct the catalytic tests for 6 h assuming time zero once the reaction temperature reaches the temperature set point (ca. 5 min). Reaction sample aliquots were periodically withdrawn through a refrigerated sample collector and filtered before analysis. Catalytic recycling tests were performed using the spent catalysts recovered by filtration from previous assays. Samples were washed in between recycling tests with methanol in an ultrasound bath for 30 min and air-dried at room temperature overnight.

### Sample Analysis

Samples from the catalytic tests were analyzed by HPLC and GC. HPLC analyses were conducted on an Agilent 1260 unit using a Shodex Asahipak NH2P-50 4E column and acetonitrile/water (80:20 vol) as mobile phase for sugar quantification. The rest of the products were quantified on a Varian CP3900 GC unit, fitted with a CPWAX-52-CB column, using *n*-decane (0.01 g·L^–1^) as the internal standard. Substrate conversion (*X*_i_) and product yields (*Y*_i_) were calculated as follows:
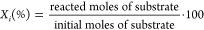
1
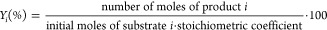
2

The stoichiometric coefficient refers to the number of carbon atoms of the starting substrate divided by the number of carbon atoms derived from the starting substrate in the considered product (Supporting Information, Table S1). In this way, product yields are calculated on the basis of the carbon atoms of the starting substrate finally incorporated into the final product.

### Recovery of GVL-Organosolv Hemicellulose

The recovery of carbohydrates from lignocellulosic biomass was carried out through a GVL organosolv fractionation process.^30^ Wood chips were treated with sulfuric acid (0.1 mol·L^–1^) in a GVL/water solution (70:30 wt %) for 1 h at 125 °C. This allowed the effective dissolution of lignin and hemicellulose, which were separated from cellulose by filtration. The resultant liquor was diluted with water to precipitate lignin, which was separated by centrifugation. The clarified solution was then treated with toluene in a continuous solvent extractor for solvents lighter than water, allowing the complete removal of GVL and furanics, which was assessed by means of HPLC analysis (HiPlex H column, H_2_SO_4_ 5 mM as an eluent at 0.6 mL·min^–1^, Refraction Index Detection). The resultant solution was then neutralized with Ca(OH)_2_, leading to the precipitation of gypsum and separation of sulfate ions. The final solution was contacted with an acidic carbon (CABOT Black Pearls 2000) to adsorb remaining organics in the solution (e.g., acid soluble lignin). The aqueous sugar solution was then frozen in liquid nitrogen and lyophilized to recover the carbohydrates. Carbohydrate analysis was conducted through the NREL/TP-510-42623 standard (Supporting Information, Table S2).

## Results and Discussion

Table 1 lists the physicochemical properties of the K-exchanged and Sn-functionalized USY and β zeolites. ICP-OES analyses confirmed the presence of tin, aluminum, and potassium in both USY and β zeolites. The presence of Al reflected the incomplete dealumination of the parent zeolites even after applying two consecutive dealumination treatments using concentrated nitric acid. The remaining Al species are more abundant in the USY than in the β zeolite, most probably because USY is featured by larger particle sizes, as evidenced by its smaller surface area as compared to the β zeolite, which results in difficulty in aluminum leaching in less accessible locations. On the other hand, tin was almost quantitatively incorporated to the zeolites (>90%) because of the high efficiency of the grafting process. Finally, potassium incorporated into the zeolites through ion exchange in Bro̷nsted acid sites was present in similar concentrations in both zeolites. This treatment allows for boosting the catalytic activity of tin sites in the retro-aldol reaction of sugar monosaccharides.

**Table 1:** Physicochemical Properties of the Prepared Catalysts

sample	[K]Sn-USY	[K]Sn-β
Al [wt %]^a^^a^	0.55	0.15
Sn [wt %]^a^	1.91	1.85
K [wt %]^a^	0.40	0.37
acid capacity [meq H^+^·g^–1^]^b^	0.60	0.70
Bro̷nsted/Lewis acid ratio^c^	9.0	0.9
*S*_BET_ [m^2^·g^–1^]^d^	688	595
*S*_μ_ [m^2^·g^–1^]^e^	459	333
*V*_t_ [cm^3^·g^–1^]^f^	0.45	0.59

a Measured by ICP-OES.

b Acid capacity calculated from NH_3_-TPD.

c Bro̷nsted to Lewis acid ratio as determined from pyridine DRIFT experiments.

d Specific surface area calculated by the BET method.

e Micropore surface area determined by the *t*-plot method.

f Total pore volume recorded at *P*/*P*_0_ = 0.95.

X-ray diffraction patterns (Supporting Information, Figure S1A) collected for the [K]Sn-functionalized catalysts correspond to FAU and BEA structures showing highly intense diffraction signals with little or no changes from the XRD patterns recorded for parent samples. This confirmed that the structures of the zeolites were well preserved during the synthesis of the catalysts. As for the textural properties, both zeolites displayed type-I argon adsorption–desorption isotherms, typical of microporous materials, featured with small H3 ([K]Sn-β) and H4 ([K]Sn-USY) hysteresis loops, which are caused by the adsorption of argon within the interparticular voids of the catalyst samples (Supporting Information, Figure S1B). Besides, both materials displayed some adsorption capacity in the medium pressure range (*P*/*P*_0_ = 0.5–0.7), which is caused by the presence of mesopores in these materials. Pore size distributions calculated through NLDFT methods (Supporting Information, Figure S1C) confirmed the existence of mesoporosity in these materials, its origin most likely being the dealumination step of parent zeolites. In addition, the β material displayed a considerably high external surface area, as determined through the application of the *t*-plot method, due to a relatively small particle size.^31^ Scanning electron microscopy demonstrated that the β material was constituted by agglomerates of nanosized zeolite crystals, whereas the USY zeolite displayed much larger crystal sizes (Supporting Information, Figure S3). Acid properties, evaluated by means of ammonia TPD experiments and DRIFT analyses using pyridine as a molecular probe, confirmed the existence of both Lewis and Bro̷nsted-type acid sites in the tin-functionalized materials. Pyridine DRIFT spectra of the zeolites during the different stages of the synthesis procedure are depicted in Figure S3 (Supporting Information). All of the spectra displayed the characteristic bands attributable to different vibration bands of pyridine adsorbed onto Bro̷nsted and Lewis acid sites. Thus, signals at 1634 and 1544 cm^–1^, which are conventionally ascribed to H-bonded pyridine in strong Bro̷nsted acid sites (BAS), were distinguishable in most of the tested materials. On the other hand, the signals detected at 1614 and 1452 cm^–1^ are due to the adsorption of pyridine onto Lewis acid sites (LAS), and these were present in all the recorded spectra. Finally, the signal located at 1490 cm^–1^ is a combination of pyridine adsorbed onto Lewis and Bro̷nsted acid sites. Several differences can be found in the proportion of Lewis to Bro̷nsted acid sites between the USY and β zeolites. Thus, in the case of the faujasite material, the dealumination and tin incorporation resulted in a strong decrease in Lewis and Bro̷nsted acidity, as can be concluded from the marked reduction in the intensity of all the pyridine vibration bands. The removal of both intraframework and extraframework aluminum species, associated with Bro̷nsted and Lewis acid sites, respectively, in the USY parent material is the main reason for the reduction of the acidity. Although the ion exchange step led to a reduction in acid capacity in the [K]Sn-USY material, it was nonetheless unable to remove all the Bro̷nsted acidity, as is easily inferred from the presence of pyridine vibration bands ascribed to BAS. This is directly linked to the aluminum species remaining attached to the zeolite after the synthesis of the material but is also probably due to a high population of silanol groups on the catalyst surface, which are not neutralized by ion exchange with potassium but provide Bro̷nsted acidity. This is probably the main reason for the very high Bro̷nsted to Lewis acid ratio found in [K]Sn-USY even after the ion exchange of acid protons with potassium ions (B/L = 9.0). On the other hand, the β material exhibited a different behavior, mainly because the parent material possesses significantly different acid properties. The starting H-β showed much lower Lewis acidity than H-USY due to the low amount of extraframework aluminum sites.^32^ The removal of intraframework aluminum and the incorporation of tin species to the BEA structure depressed the Bro̷nsted to Lewis acid ratio, and the ion exchange of acidic protons by potassium cations decreased this ratio even more (B/L = 0.9), as it corresponds to a material with a lower amount of remaining aluminum sites. These differences between both materials are expected to exert a notable influence on the catalytic activity of both materials.

**Figure 1:**
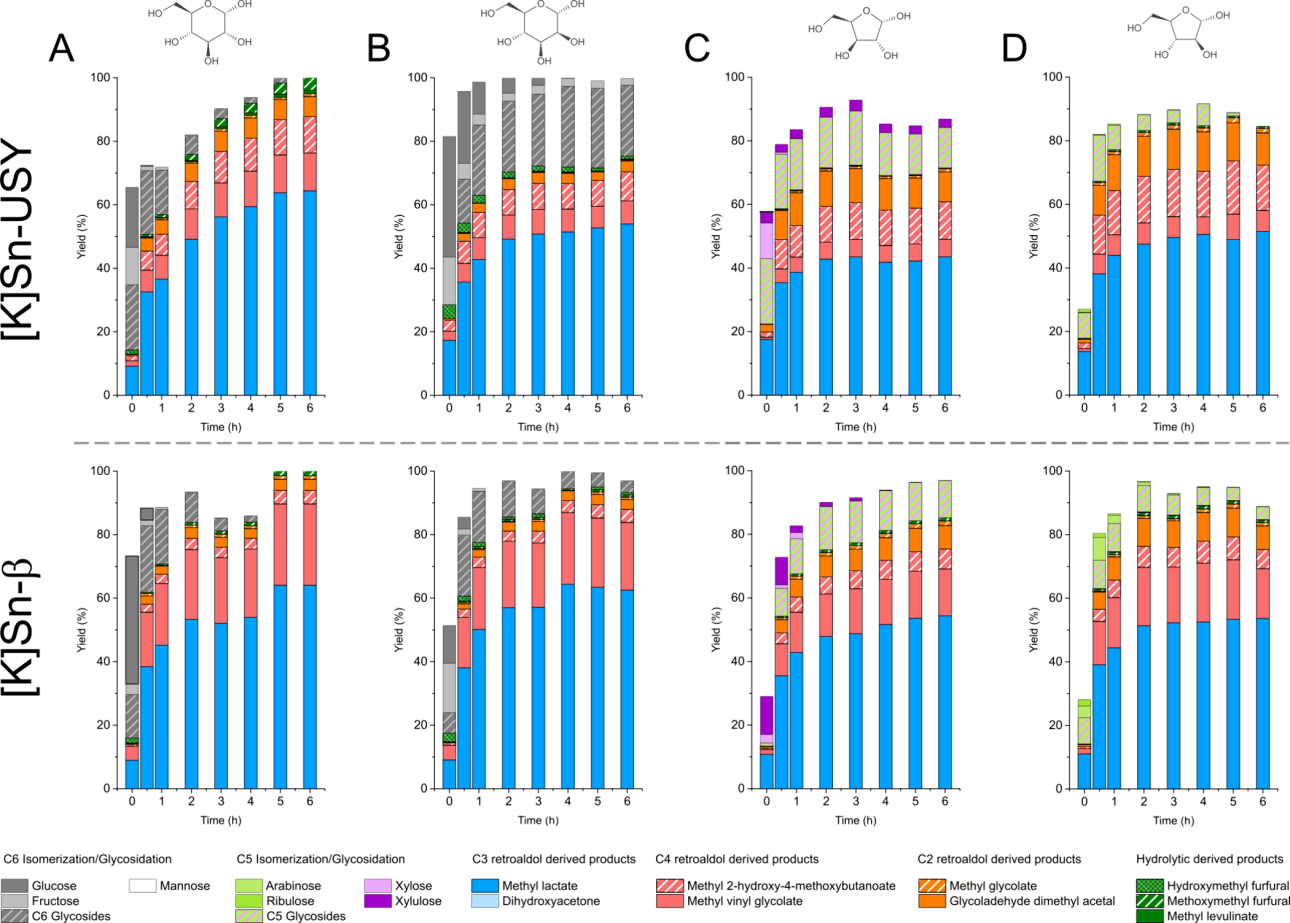
Product distribution obtained with [K]Sn-USY and [K]Sn-β catalyst in the transformation of hemicellulose monosaccharides (A, glucose; B, mannose; C, xylose, and D, arabinose) in methanolic media. Reaction conditions: monosaccharide concentration = 48 g·L^–1^; catalyst loading = 0.75 g; reaction volume = 75 mL; 150 °C; 13 bar (autogenous pressure).

Figure 1 depicts the results achieved in the transformation of glucose, mannose, xylose, and arabinose in the presence of [K]Sn-USY and [K]Sn-β zeolites at 150 °C for 6 h. These monosaccharides were selected as reaction substrates because of their abundance in hemicellulose hydrolysates obtained from different plant species.^33^ Both zeolites promoted the transformation of all the tested carbohydrates, reaching a complete substrate conversion after a few hours and yielding methyl lactate (MLA) as the main reaction product. Other products detected in the reaction media included C2 methyl glycolate (MG) and glycolaldehyde dimethyl acetal (GADMA), C4 sugar derived products methyl vinyl glycolate (MVG) and methyl 2-hydroxy-4-methoxybutanoate (MMHB), and some other chemicals in minor quantities like 5-hydroxymethylfurfural (HMF), 5-methoxymethylfurfural (MMF), and methyl levulinate (MLE). No products derived from the incorporation of methanol, the reaction solvent, to the carbon backbone of final products were observed. The presence of such a variety of bioproducts confirms the existence of different reaction pathways as depicted in Scheme S1. The transformation of sugar monosaccharides proceeds through their isomerization from an aldose to a ketose form followed by the retro-aldol cleavage of the existing carbohydrates to produce C2, C3, and C4 sugars, finally leading to the formation of the described products. Alternatively, hydrolytic pathways involving the dehydration of sugars lead to the formation of HMF, its methyl ether (MMF), and its hydrolysis derivative, methyl levulinate.

Regarding the comparison of product distributions, MLA emerged as the main product in all of the cases, albeit with some differences between hexoses and pentoses. Considering that the retro-aldol splitting of a hexose yields two trioses, whereas a pentose is transformed into a single triose and glycolaldehyde (Supporting Information, Scheme S1), the productivity of MLA from C5 sugars should be half of that achieved from hexoses. The small differences between hexoses and pentoses as substrates for MLA production suggest the existence of parallel transformations favoring the formation of MLA from pentose sugar substrates. In this sense, Dusselier et al.^34^ demonstrated that tin-functionalized zeolitic materials are able to promote aldol condensation of GA to larger sugars, facilitating the indirect transformation of GA into MLA. Moreover, the study on the treatment of GA with Sn-functionalized silicates evidenced the feasibility to transform GA into tetrose and hexose sugars, with distinct selectivity depending on the pore size of the used catalyst.^21,24^ In this regard, differences between β and USY zeolites are expected for MLA productivity; however, the results indicate that variations are observed in the distribution of secondary products. Thus, both zeolites provided C2—methyl glycolate and GADMA—and C4 products—MMHB and MVG—particularly when treating pentoses. These results indicate that the tested [K]Sn-functionalized zeolites feature a high catalytic activity in retro-aldol cleavage of sugars and also a high activity in C–C self-coupling of GA to produce, at least, tetrose sugars. Regarding the differences between zeolites, the USY material provided high amounts of methyl glycolate and GADMA when starting from pentoses (15–30% combined yield, depending on the sugar), whereas the yield to C2 products achieved in the presence of the β zeolite was much lower (<10%). This might be linked to the higher proportion of weak BAS in the faujasite material, which catalyzes the formation of the methyl hemiacetal and acetal derived from glycolaldehyde, thus leading to methyl glycolate and GADMA, respectively. This pathway prevents the condensation of GA to C4 products, which, in turn, are more abundant when using the [K]Sn-β zeolite. As for the C4 products, the USY-based material yielded MMHB as the main C4 product, whereas [K]Sn-β produced MVG. This difference might be ascribed to the different pore sizes of both zeolites, as [K]Sn-USY (0.74 × 0.74 nm) seems to easily accommodate MMHB, but the smaller pore size of the β zeolite (0.76 × 0.64 nm) would prevent its formation, favoring a dehydration of the intermediate product to provide MVG instead (Supporting Information, Scheme S1). This suggests a size exclusion effect when the BEA zeolite is used, which leads to a higher selective material for MVG production. Interestingly, α-hydroxy butanolide, a C4 product obtained when treating glycolaldehyde in the presence of Sn-β catalysts,^24^ was not detected within the reaction media. This difference might be ascribed to the different reaction media and temperature conditions, which, in our case, may favor the dehydration of methyl-2,4-dihydroxy butanoate, an intermediate produced from tetroses.

**Figure 2:**
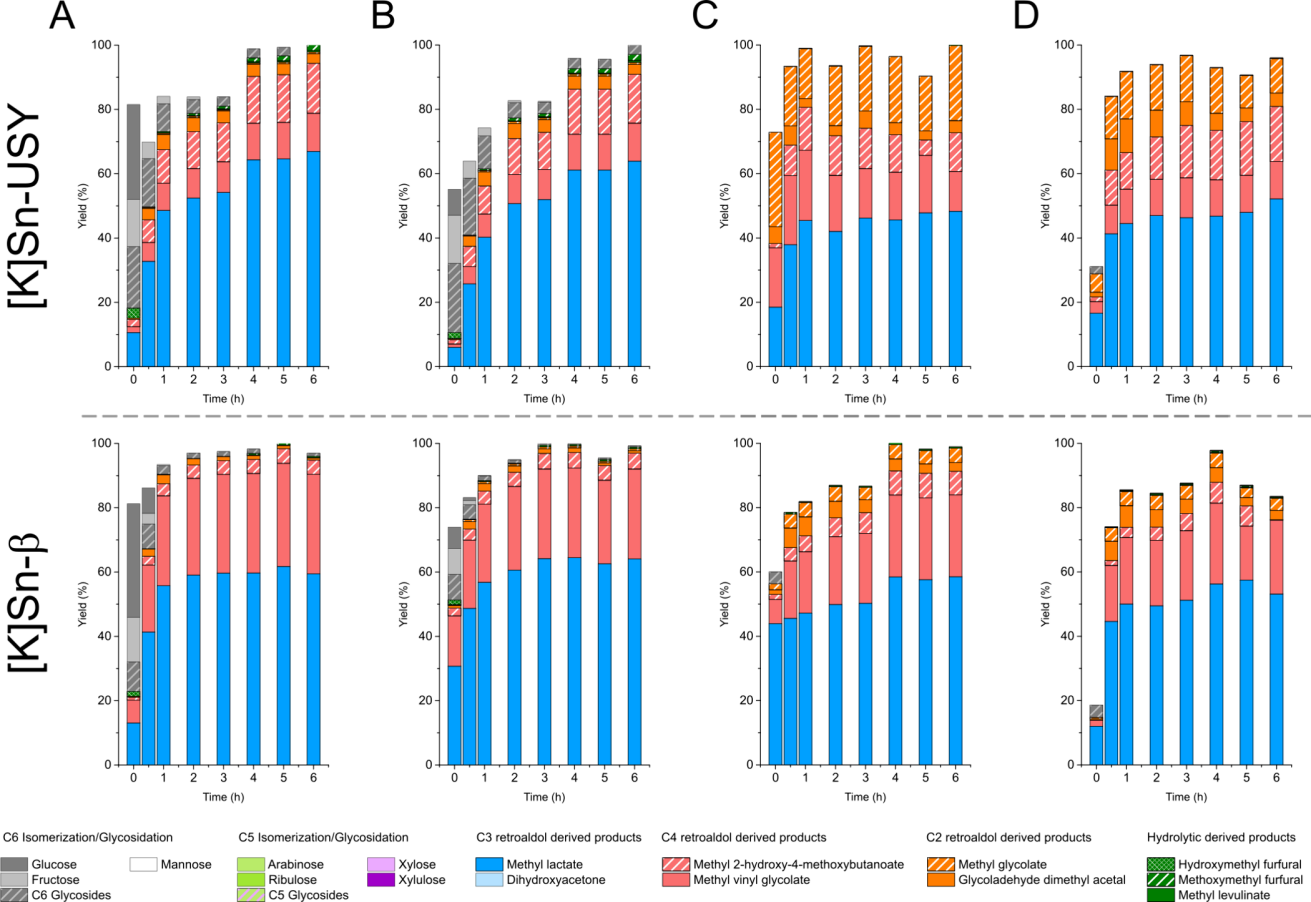
Product distribution obtained with the [K]Sn-USY and [K]Sn-β catalyst in the transformation of hemicellulose monosaccharides (A, glucose; B, mannose; C, xylose and D, arabinose) in methanol/water (96:4 wt %) media. Reaction conditions: monosaccharide concentration = 48 g·L^–1^; catalyst loading = 0.75 g; reaction volume = 75 mL; 150 °C; 13 bar (autogenous pressure).

The catalytic experiments starting from the same sugars, glucose, mannose, xylose, and arabinose, were repeated in the presence of small quantities of water. The purpose of these experiments was to boost the activity of the tin catalysts in aldol-condensation reactions and enhance MLA productivity. Water has been described to generate Bro̷nsted acid sites on Sn-functionalized zeolites,^35^ which promotes the activation of aldehyde groups, thereby favoring C–C coupling reactions.^36^ In this way, adding some water to the reaction media aims to produce a higher proportion of MLA when starting from pentoses. Results from these catalytic tests are illustrated in Figure 2.

Substrate conversion was faster in the presence of small amounts of water, regardless of the starting sugar, probably because of a collection of different reasons, including the enhancement of the solubility of the sugar substrates in the reaction media. Sugars are soluble in methanol at high temperatures, but the addition of small quantities of water enhances this solubility. However, the reaction acceleration was much more evident for the β zeolite than for [K]Sn-USY under the same reaction conditions. Reasons for this boosted catalytic activity of the Sn-functionalized catalysts in the presence of water could be ascribed to the interaction of water with the tin sites, leading to the formation of a higher proportion of open Sn sites,^37^ which are known to be much more active than their closed counterparts.^38^ Regarding the product distribution, once again, methyl lactate emerged as the main product obtained with both zeolites for all the tested substrates. The presence of water enhanced the production of methyl lactate but only when treating pentose sugars, as expected. This effect can be attributed to the promotion of aldol condensation of small sugar intermediates. Regarding the rest of the products, the very low amount of C2 products—GADMA and methyl glycolate—obtained in these experiments provides compelling evidence for the beneficial influence of water molecules in the promotion of the self-aldol condensation of the evolving GA to C4 and C6 intermediates. Interestingly, unlike the [K]Sn-β catalyst, [K]Sn-USY produced some methyl glycolate when treating pentoses, suggesting that the ability of the USY zeolite to promote aldol condensation is lower than that of the β catalyst. Other differences between the tested zeolites are related to the product distributions. [K]Sn-USY yielded two C4 products— MVG and MMHB—in almost equimolar yields (∼10% each) together with methyl glycolate. On the contrary, [K]Sn-β produced similar product distributions regardless of the starting substrate. It yielded methyl lactate and methyl vinyl glycolate as the main products, accounting for >90% of the starting substrate when processing hexose and 72–75% when starting from pentoses. The product distributions obtained in the presence of the [K]Sn-β catalyst evidence the higher selectivity of this structure for C3 and C4 sugars as product intermediates. This could be related to the smaller pore size of the β zeolite, exerting a more pronounced confinement effect than the faujasite material that, in turn, imposes weaker restrictions to reaction intermediates, allowing the formation of a wider distribution of products.

The activity of [K]Sn-USY and [K]Sn-β zeolites in the transformation of different sugars into methyl lactate has been completed by assessing the reusability of both catalysts in the same transformation in both MeOH and MeOH + H_2_O as reaction solvents. The results of these tests are depicted in Figures S4 and S6, respectively. Overall, both the USY and β zeolite exhibited a significant decline in catalytic activity when tested in methanol as the reaction solvent, yielding less than half of the substrate conversion and product yields achieved with fresh catalysts. Analysis of spent catalysts using ICP-OES indicated that most (95%) of the initial tin and potassium contents in the zeolites were well preserved. However, thermogravimetric analysis (Supporting Information, Figure S5) revealed the presence of significant amounts of organic deposits on the spent catalysts ([K]Sn-USY: 17 wt % for mannose and 17 wt % for arabinose; [K]Sn-β: 28.5 wt % for mannose and 16 wt % for arabinose). These results suggest that the organic deposits accumulate inside the porous structure of the catalysts, impeding access to catalytic sites and causing observed catalyst deactivation. The origin of these deposits could be side products with low solubility in methanol. Results from reusability tests conducted in methanol/water (Supporting Information, Figure S6) indicate that the addition of small quantities of water to the reaction media enhanced the reusability of both zeolites when hexoses were used as reaction substrates, yielding similar substrate conversions and product distributions to those of the fresh catalyst samples. These results do not imply that the stability of the catalysts is enhanced because water can damage the zeolite catalysts, but the beneficial effect of water in the prevention of catalyst deactivation by organic deposits has a more significant effect than the eventual activity decay due to damage to the zeolites, if occurring, in the two consecutive catalytic tests. However, the results were different for pentoses as substrates, especially in terms of product distributions. In this case, the amount of C2 products, GADMA and methyl glycolate, increased substantially together with the carbon balance in the catalytic tests, suggesting that side reactions related to C2 moieties like glycolaldehyde conducting to unknown products are minimized in the presence of water.

The thermogravimetric analysis of the spent catalysts after the recycling tests in MeOH + H_2_O revealed that the presence of organic deposits in these materials was much lower than when used in methanol ([K]Sn-USY: 16 wt % for mannose and 10 wt % for arabinose; [K]Sn-β: 6 wt % for mannose and 6 wt % for arabinose, Supporting Information, Figure S7). This result indicates that most of the organic deposits detected in the catalysts used in methanol might show a low solubility in the bare alcohol, but the addition of small amounts of water might mobilize them because of either enhancing their solubility in the reaction media or preventing their formation.

To get a better insight into the transformation of both types of sugar monosaccharides (hexoses and pentoses) into methyl lactate, dihydroxyacetone (DHA) and glycolaldehyde (GA), the two main intermediates in the studied transformation of carbohydrates (specially pentoses), were tested in methanol + H_2_O (4 wt %).

**Figure 3:**
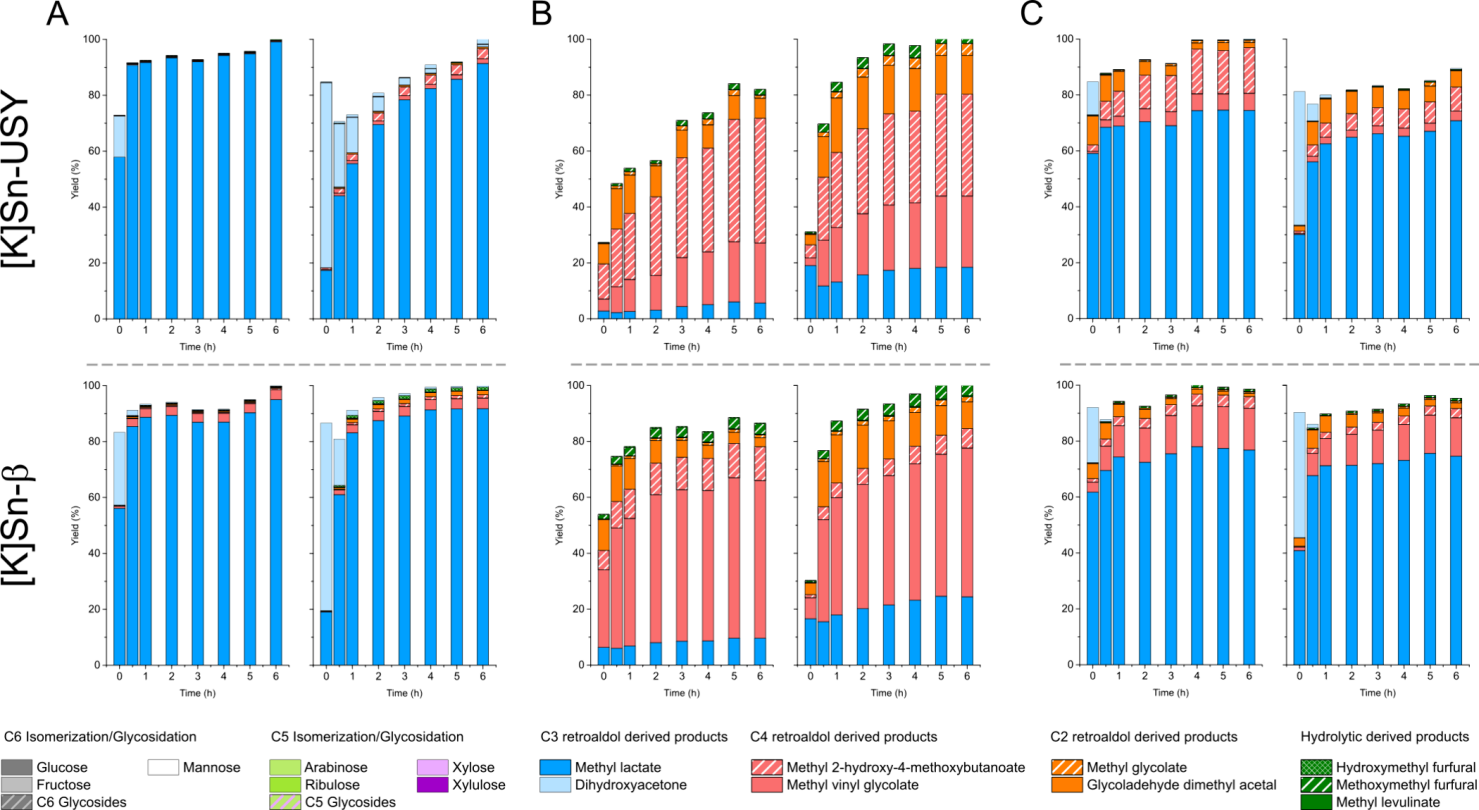
Product distributions obtained with [K]Sn-USY and [K]Sn-β catalyst (fresh catalyst, left; first reuse, right) in the transformation of reaction intermediates (A, dihydroxyacetone (C3); B, glycolaldehyde (C2), and C, dihydroxyacetone and glycolaldehyde equimolar mixture) in methanol/water (96:4 wt %) media. Reaction conditions: dihydroxyacetone concentration = 28.8 g·L^–1^; glycolaldehyde concentration = 19.2 g·L^–1^; catalyst loading = 0.75 g; reaction volume = 75 mL; 150 °C; 13 bar (autogenous pressure).

Figure 3A depicts the results achieved in the catalytic transformation of DHA solutions in the presence of [K]Sn-USY and [K]Sn-β zeolites. DHA was converted with high efficiency into methyl lactate in the presence of both zeolites when using fresh catalyst samples, providing almost quantitative MLA yields above 95% at 6 h. The analysis of reaction media by HPLC allowed detecting several hexoses (dendroketose, sorbose, and fructose) coming from the aldol condensation of the starting triose but only during the early beginning of the reaction because these were readily converted after 10 min of reaction. The existence of these hexoses confirmed that both catalysts can drive the aldol condensation of DHA during the first steps of the catalytic assays. Dendroketose can evolve from the aldolic condensation of DHA, whereas sorbose and fructose, being linear hexoses, must be produced from the coupling of DHA with glyceraldehyde. This implies a rapid isomerization of DHA in the presence of the [K]Sn-functionalized zeolites, most probably occurring during the warm-up of the reaction setup. However, all three ketohexoses only appear during the early stages of the catalytic tests, and then they are readily consumed, probably through a backward retro-aldol reaction to produce DHA once again. In this regard, slight differences were observed between both catalysts in terms of product distribution, with the [K]Sn-β zeolite being able to promote a faster transformation of the starting DHA. Nevertheless, this zeolite also provided quantifiable amounts of methyl vinyl glycolate (∼5%), which was detected in only negligible concentrations when using the [K]Sn-USY zeolite. This result makes evident the formation of C4 fragments from the starting C3 DHA, suggesting the superior performance of the β zeolite in both aldol and retro-aldol reactions, at least better than USY zeolites. Recycling tests performed in the transformation of DHA revealed a high level of reusability of both zeolites with little decrease in the production of methyl lactate when reusing the catalysts, although the most evident effect is the reduction in the reaction rate as determined from the MLA apparition rate. The high catalyst reusability might be related to the very high selectivity of the transformation of DHA into MLA, with almost quantitative conversion, which does not lead to the formation of significant amounts of side products, preventing the deactivation of the catalysts.

Regarding the conversion of GA (Figure 3B), commercially available glycolaldehyde dimer was selected as a reaction substrate, a suitable chemical liable to be used as a GA source.^24^ The identified products achieved in these tests included, similarly to that previously observed for tests with DHA, the formation of large sugars during the early beginning of the reaction, which included a mixture of tetroses (threose, erythrose, and erythrulose). These sugars were accompanied by the formation of GADMA, MMHB, MVG (C2 and C4 products), and methyl lactate in quite high yields. These results evidence that aldol condensation of glycolaldehyde is taking place in the presence of tin-functionalized zeolites, confirming our conclusions about the high activity shown by these catalysts in the production of MLA when treating pentoses. Because the formation of MLA requires C3 intermediates like DHA or GLA as product intermediates, we postulate that the condensation of three units of GA to form a hexose molecule followed by its subsequent cleavage into two trioses is the most plausible reaction pathway to achieve methyl lactate from glycolaldehyde. As for the differences found between [K]Sn-USY and [K]Sn-β as catalysts, the latter provided higher product yields in a shorter time, indicating a faster performance as a catalyst. Regarding the product distributions, here again, the USY-based zeolite provided higher yields of MMHB, whereas the β-zeolite provided a higher selectivity for MVG. Recycling tests revealed no catalyst deactivation; in fact, the opposite was observed, as higher yields were achieved for all of the detected reaction products using both zeolites. [K]Sn-β produced large amounts of methyl lactate (∼25%) and C4 products (∼55%), higher than those of the faujasite zeolite (17 and 50%, respectively). These results prompt a higher catalytic activity of the [K]Sn-β catalyst in aldol condensation reactions, as previously stated. Besides, the high reusability of the catalysts suggests that the formation of organic deposits onto the surface of the catalysts leading to their deactivation is not related to C2 moieties. Regarding the observed acceleration in the catalytic activity, this phenomenon could be caused by the modification of the catalytic sites due to the presence of water. As previously stated, water favors the opening of tin sites, thus enhancing the Lewis acidity of tin sites, but also promotes a higher hydration degree, which modifies the activity and selectivity of tin sites because of the modification of the Bro̷nsted acidity as elsewhere reported.^39^

Finally, equimolar mixtures of DHA and GA, simulating the hypothetical combination derived from a pentose, were also treated as reaction substrates (Figure 3C). The primary product obtained in these experiments was MLA, reaching yields of around 70% after 1–2 h. The rest of the products included GADMA, MVG, and MMHB, exhibiting product distributions consistent with those observed when treating DHA and GA, separately. The MLA yield achieved in these experiments, quite over that expected (ca. 55%), suggests some interaction between the transformations of DHA and GA. In this regard, we postulate that because DHA must be dehydrated on its way to MLA, this transformation might lead to higher hydration degrees on tin sites, boosting their catalytic activity in aldol condensation reactions and enhancing the productivity of MLA from GA. However, no products showing C5 backbone were detected, indicating the absence of side reactions involving the C2 and C3 components. Nevertheless, TGA analyses revealed similar contents of organic deposits in the spent catalysts to those found in samples used for converting monosaccharides. To ascertain their nature, spent catalyst samples corresponding to the [K]Sn-β zeolite used with DHA and GA were analyzed through ^13^C and ^1^H MAS NMR and washed with deuterated chloroform to analyze the extracted compounds (Supporting Information, Figure S8). The main resonances correlate well with those coming from the hydroxyesters obtained as final products (ML, MVG, MMHB) to the reaction solvent and the internal standard used for quantification through gas chromatography. In this way, no evidence of the presence of extractable bulk products was detected in the spent catalysts, confirming the beneficial effects of the addition of water on the prevention of carbon deposition on the surface of the catalysts.

**Figure 4:**
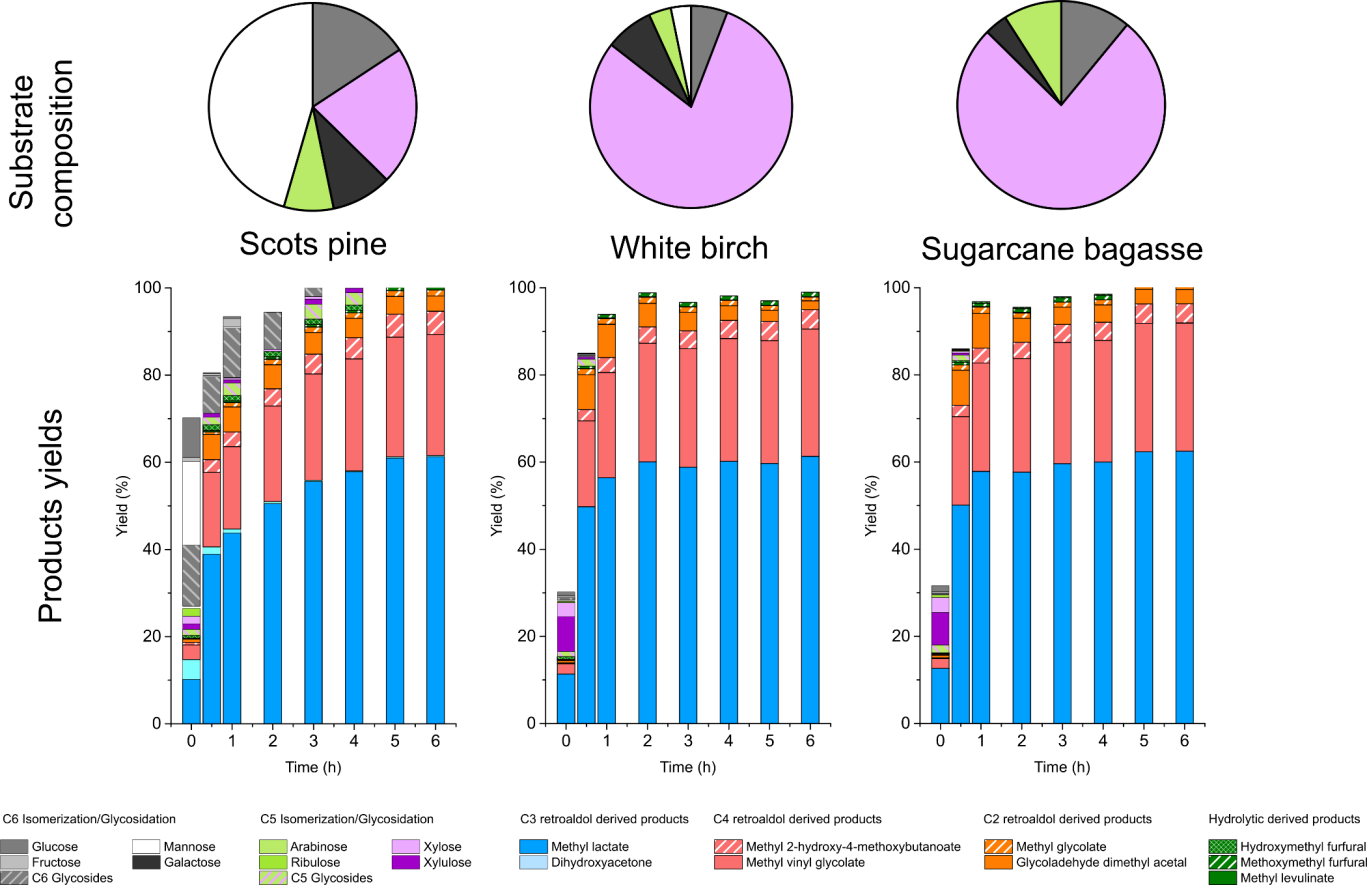
Starting composition and product distribution obtained in the transformation of several sugar mixtures representing hemicelluloses from Scots pine, white birch, and sugar cane bagasse in the presence of [K]Sn-β. Reaction conditions: initial total sugar loading: 48 g·L^–1^; catalyst loading = 0.75 g; reaction volume = 75 mL; 150 °C.

The very high catalytic activity, as well as the higher product selectivity toward a limited range of α-hydroxymethyl esters, shown by the [K]Sn-β zeolite when treating hexoses, pentoses, and small sugars, prompted us to test this catalyst in the transformation of complex sugar mixtures. For this purpose, we used three different sugar mixtures representing hemicellulose hydrolysates obtained from three different biomass feedstock: Scots pine, white birch, and sugar cane bagasse. The compositions of these mixtures were determined as detailed in the Supporting Information. Scots pine was selected because it is one of the few sources of C6-rich hemicelluloses, showing a total content of 65% of hexoses (mainly mannose and galactose),^40^ with the rest of the carbohydrate content being xylose and arabinose. On the other hand, white birch and sugar cane bagasse were selected because of the opposite reason, because associated hemicelluloses mainly consist of pentoses (>80%), favoring their use as feedstock to produce a single product like furfural.^30,41,42^Figure 4 depicts the composition and product distribution obtained for the different complex sugar mixtures used as starting feedstocks in the production of methyl lactate in the presence of the [K]Sn-β zeolite. Detailed information on the composition, extraction efficiency, and sugar recovery found for the different GVL-organosolv hemicellulose hydrolysates for different woods can be found in Table S2 (Supporting Information). Results from the catalytic tests performed with [K]Sn-β provide evidence of the extraordinary catalytic activity, in terms of mass balance and overall product yield, of the tested zeolite to drive the simultaneous transformation of hexoses and pentoses into methyl lactate and methyl vinyl glycolate. The main differences observed in the transformation of the tested sugar mixtures are concentrated in the early beginning of the assays, because a faster transformation of the pentose-rich sugar mixtures is detected, in good agreement with the observed behavior when treating pure monosaccharides. Nevertheless, the obtained results reflect a high similarity in the product distributions obtained from sugar mixtures, showing very different carbohydrate profiles. Nearly complete conversion of the starting sugar mixtures into methyl lactate (∼60% yield) and methyl vinyl glycolate (∼30% yield), with a low production of side products, was achieved for all the tested feedstock regardless of their composition. These results reflect an outstanding catalytic performance of the [K]Sn-β zeolite in both retro-aldol and aldol reactions of sugars so that the starting carbohydrates (“large” pentoses and hexoses) are initially split into small sugars (glycolaldehyde and dihydroxyacetone or glyceraldehyde), which are then combined to produce C3 and C4 carbon intermediates, finally yielding ML and MVG. The similarity in the product distribution obtained from different sugar mixtures and the absence of large sized products (e.g., MMHB) suggest that the selectivity of the transformation is conditioned by the high catalytic activity of tin sites in both retro-aldol and aldol reactions of sugars, as well as by the pore size of the zeolite, which conditions the size of the evolving products arising from aldol condensation pathways, as previously reported.^25^ Considering these results, it becomes evident that the β structure offers numerous advantages for treating complex mixtures of sugars for the production of α-hydroxyesters, providing a lower number of products of commercial interest.

**Figure 5:**
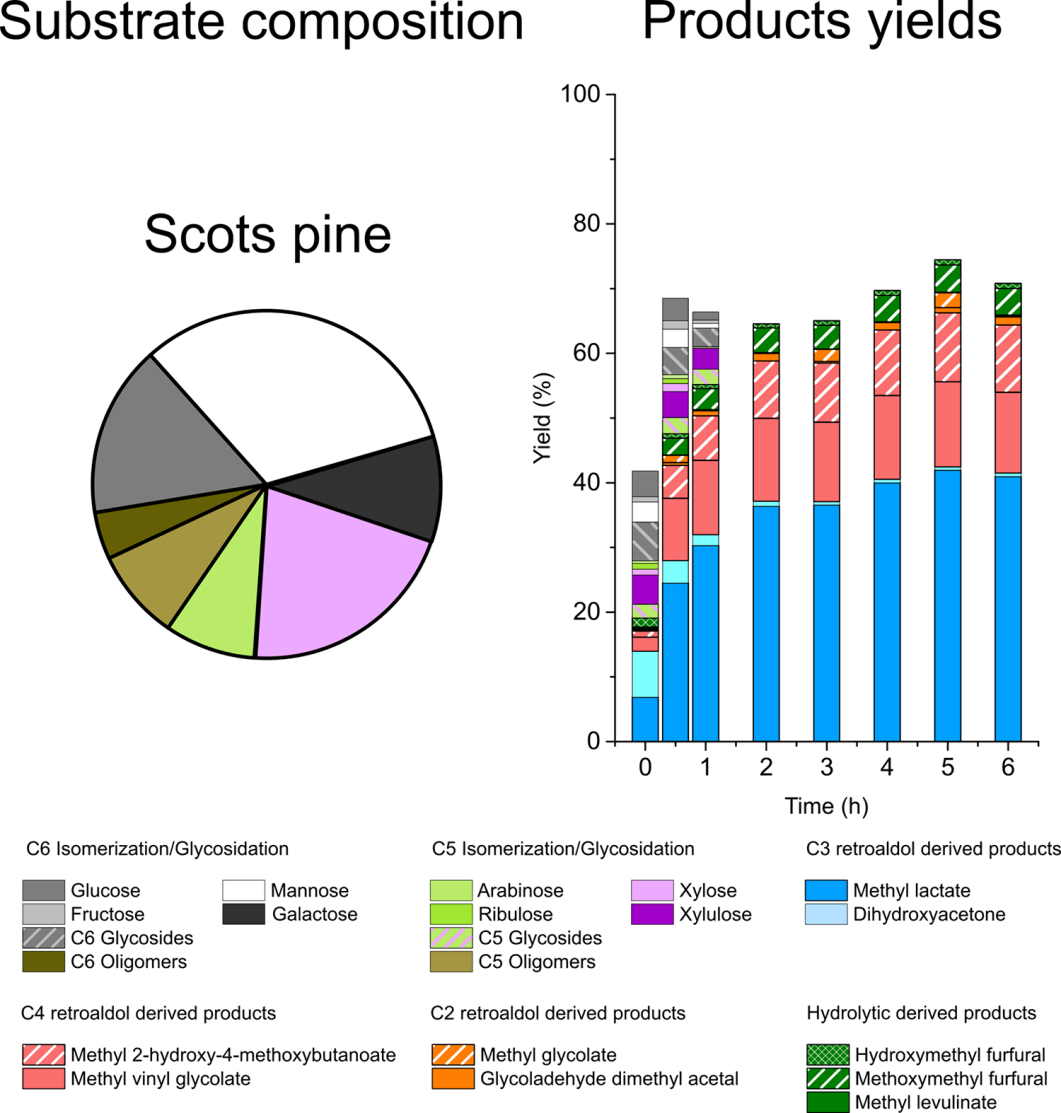
Product distribution obtained from the transformation of methanolic solutions of GVL-organosolv Scots pine hemicellulose in the presence of [K]Sn-β. Reaction conditions: initial total sugar loading: 48 g·L^–1^; catalyst loading = 0.75 g; reaction volume = 75 mL; 150 °C. The composition of the starting sugar mixture is listed in Table S2 (Supporting Information).

To further advance the assessment of the activity and versatility of K-exchanged tin-functionalized β zeolite, a blend of sugars sourced from Scots pine hemicellulose obtained through an organosolv method was used as the initial raw material. The sugar mixture comprises a range of monosaccharides, encompassing hexoses like mannose, glucose, and galactose as well as pentoses like xylose and arabinose. Additionally, a fraction of oligosaccharides is also present, which differentiates it from the hemicellulose mimic solution tested previously. The origin of these oligosaccharides is probably in the incomplete hydrolysis of larger hemicellulose carbohydrates, which remain soluble oligosaccharides. These carbohydrates contain both hexoses and pentoses; however, these were expressed as pentosan and hexosan oligosaccharides for clarity. The outcomes of treating this feedstock in the presence of [K]Sn-β zeolite are depicted in Figure 5.

The transformation of Scots pine GVL-hemicellulose in the presence of [K]Sn-β produced quite low yields of methyl lactate and MVG as compared to those achieved from synthetic sugar mixtures mimicking the same feedstock. This discrepancy is likely attributed to the presence of several components inherent in real hemicellulose, including the mentioned sugar oligomers and some other impurities like metal cations (Supporting Information, Table S3). Thus, sugar oligomers, because of their larger size, cannot be processed in the same manner as monosaccharides, as they cannot diffuse inside the pores of the tested zeolite and thus remain unconverted after the catalytic tests. On the other hand, a high concentration of cations, especially alkaline and alkaline-earth cations, produces the deactivation of the tin sites if excessive amounts of these metal cations are present in the reaction media, eliminating their Bro̷nsted and Lewis acid catalytic activity, as previously reported.^29^ However, and in spite of the presence of these impurities conditioning the catalytic activity of [K]Sn-β, an outstanding yield toward methyl lactate of approximately 40% was achieved together with some MVG and MMHB. This resulted in a cumulative yield of the three α-hydroxyesters accounting for 65% after 6 h of reaction. Moreover, if the product distribution achieved when treating genuine and simulated hemicelluloses is compared, the few differences observed between the results of both catalytic tests can be ascribed to the presence of oligosaccharides, which reduce the maximum achievable product yields to the values obtained from real hemicellulose. These results confirm the very good catalytic performance of potassium-exchanged tin-functionalized β zeolite in the transformation of complex mixtures of sugars, even those directly obtained from natural sources, to provide a narrow range of bioproducts of commercial interest, including methyl lactate and other α-hydroxy esters.

## Conclusions

K-exchanged, Sn-bearing zeolites with BEA and FAU topologies promote the transformation of a wide collection of several carbohydrates into methyl lactate under moderate reaction conditions. These zeolitic catalysts display not only excellent catalytic activity but also high reusability if the reaction medium is properly tuned by the addition of water to minimize the deposition of organics onto the surface of the catalysts. Insights in the conversion of product intermediates from carbohydrates to methyl lactate, dihydroxyacetone, and glycolaldehyde revealed the ability of the tested zeolites to also drive the aldol condensation of small sugars, yielding tetroses and hexoses, a transformation in which the [K]Sn-β zeolite seemed superior to its USY counterpart. This pathway allowed for converting both hexoses and pentoses into methyl lactate and some other α-hydroxyesters (MVG, MMHB) in a highly efficient manner. To validate this potential, [K]Sn-β was tested in the treatment of several sugar mixtures mimicking hemicelluloses from different biomass feedstock. Results revealed that the tested catalyst was able to convert both hexoses and pentoses into a limited set of α-hydroxy esters with commercial interests, including methyl lactate and methyl vinyl glycolate, yielding a similar product distribution regardless of the starting composition of the sugar mixture. This feature was attributed to the ability of [K]Sn-β to drive both aldol and retro-aldol condensation of sugars in a highly efficient manner. Finally, a real hemicellulose mix of sugars, obtained from Scots pine wood through a GVL-based organosolv fractionation method, was tested as a substrate. Reaction results revealed that the Sn-functionalized β zeolite was able to convert this complex mixture of sugars with high selectivity for a limited number of products of commercial interest.

## Supporting Information Available

Detailed reaction scheme, results from the characterization of the zeolite catalysts, and results from additional catalytic tests and from the characterization of spent catalyst samples (PDF)
