# Supplementary material for: Production of Methyl Lactate with Sn-USY and Sn-β: Insights into Real Hemicellulose Valorization
Source: ACS Sustain Chem Eng. 2024 Feb 6;12(7):2771–82. doi: 10.1021/acssuschemeng.3c07356 (PMC10880092; doi:10.1021/acssuschemeng.3c07356)
Supplement: Supplementary file 1 — sc3c07356_si_001.pdf [file sc3c07356_si_001.pdf]

SUPPORTING INFORMATION

Production of methyl lactate with Sn-USY and Sn-β: insights into real hemicellulose valorization

*Jose M. Jiménez-Martin,† Miriam El Tawil-Lucas,† Maia Montaña,† Maria Linares,† Amin Osatiashtiani,⚬ Francisco M. Vila,‡ David Martín Alonso,‡ Jovita Moreno,† Alicia García,† and Jose Iglesias†,□,**

† Chemical & Environmental Engineering Group. Universidad Rey Juan Carlos. C/ Tulipan s/n, 28933. Madrid, Spain.

⚬ Energy & Bioproducts Research Institute (EBRI), College of Engineering and Physical Sciences, Aston University, Aston Triangle, Birmingham B4 7ET, United Kingdom

‡ Energy and Sustainable Chemistry (EQS) Group. Institute of Catalysis and Petrochemistry, CSIC, C/ Marie Curie 2, Campus de Cantoblanco. 28049 Madrid, Spain

□ Instituto de Tecnologías para la Sostenibilidad. Universidad Rey Juan Carlos. C/ Tulipan s/n,

28933. Madrid, Spain.

Number of pages: 17 (from S1 to S17)

Number of tables: 3

Number of reaction schemes: 2

Number of figures: 8

CONTENTS

REACTANTS & REAGENTS

TABLES

Table S1. Stoichiometric coefficients considered in the calculation of product yields –carbon basis– as a function of the starting substrate.

Table S2. Composition of hemicellulose hydrolysate recovered from different biomass sources as determined through NREL/TP-510-42623 standard. Extraction and recovery efficiencies for pentoses and hexoses.

Table S3. Impurities and their concentration found in GLV-organosolv Scots Pine hemicellulose hydrolysate.

SCHEMES AND FIGURES

Scheme S1. Detailed transformation pathways occurring in the treatment of hemicellulose monosaccharaides in the presence of [K]Sn-USY and [K]Sn-β zeolites in methanol media.

Scheme S2. Simplified reaction scheme with the principal transformations and products in the transformation of hemicellulose monosaccharaides in the presence of [K]Sn-USY and [K]Sn-β zeolites in methanol media.

Figure S1. (A) Argon adsorption-desorption isotherms, (B) pore size distribution and (C) DR-UV-Vis spectra comparison for both Sn-USY and Sn-β catalyst.

Figure S2. SEM micrographs collected for [K]Sn-USY (left) and [K]Sn-β (right) zeolites at different magnifications.

Figure S3. DRIFT spectra of pyridine adsorbed on tested zeolites (parent, Sn-functionalized, and K-exchanged) with faujasite and BEA structures.

Figure S4. Product distribution obtained in the second use of Sn-USY and Sn-β catalyst in the transformation of hemicellulose monosaccharides (A, glucose; B, mannose; C, xylose and D,

arabinose) in methanol. Conditions: monosaccharide concentration = 48 $g{\cdot}L^{-1}$; catalyst loading = 0.5g; reaction volume = 50 mL; 150°C; 13 bar (autogenous pressure); 6h reaction time.

Figure S5. Thermogravimetric analysis of spent catalyst, Sn-USY (1) and Sn-β (2), after second use in the transformation of mannose (A) and arabinose (B) in methanol.

Figure S6. Product distribution obtained in the second use of Sn-USY and Sn-β catalyst in the transformation of hemicellulose monosaccharides (A, glucose; B, mannose; C, xylose and D, arabinose) in methanol:water (96:4wt%) media. Conditions: monosaccharide concentration = 48 $g{\cdot}L^{-1}$; catalyst loading = 0.5g; reaction volume = 50 mL; 150°C; 13 bar (autogenous pressure); 6h.

Figure S7. Thermogravimetric analysis of spent catalyst, Sn-USY (1) and Sn-β (2), after second use in the transformation of mannose (A) and arabinose (B) in methanol:water (96:4wt%) media.

Figure S8. ^{13}C (A) and ^{1}H (B) NMR spectra of Sn-β spent catalyst in the transformation of dihydroxyacetone (DHA) and glycolaldehyde (GLA) (black solid lines) and the liquid-extracted compounds after washing with CD_3Cl (solid red lines).

REACTANTS & REAGENTS

Commercial USY (CBV 712, Zeolyst International) and β (CP814C*, Zeolyst International) zeolites were used as parent materials for the synthesis of Sn-USY and Sn-β materials, respectively. Nitric acid (HNO_3, Sigma Aldrich, 70%) was used in the dealumination process. Methylene chloride (CH_2Cl_2, Scharlab), tin (IV) chloride pentahydrate ($SnCl_4 \cdot 5H_2O$, 98%, Alfa Aesar), and triethylamine ($N(CH_2CH_3)_3$, NEt_3, 99%, Sigma Aldrich) were used in the metalation procedure as solvent, tin source, and grafting promoter, respectively. Potassium chloride (KCl, 99%, Sigma Aldrich) was used as alkaline cation source to produce [K]Sn-zeolites. D-(+)-glucose (GLU, 99%, Sigma Aldrich), D-(+)-mannose (MAN, 99%, Sigma Aldrich), D-(+)-xylose (XYL, 99%, Sigma Aldrich), L-(+)-arabinose (ARA, 98%, Sigma Aldrich), glycolaldehyde dimer (GLA, 99%, Sigma Aldrich) and dihydroxyacetone (DHA, 99%, Sigma Aldrich) were used in the catalytic performance test as substrates. Methanol (CH_3OH, HPLC Grade, Sharlab) and milli-Q grade deionized water were used in the catalytic performance tests as solvents. Methyl D-lactate (MLA, 99%, Sigma Aldrich), methyl glycolate (MG, 98%, Sigma Aldrich), methyl vinyl glycolate (MVG, 98.4%, Apollo Scientific), glycolaldehyde dimethyl acetal (GADMA, 98%, Alfa Aesar), methyl 2-hydroxy-4-methoxybutanoate (MMHB, 99%, BioSynth), methyl levulinate (MLE, 98%, Sigma Aldrich), methoxymethyl furfural (MMF, 95%, abcr GmbH) and 5-hydroxymethylfurfural (HMF, 98%, abcr GmbH) were used in the preparation of standards stock solutions for the calibration of the gas chromatography unit, using n-

decane ($C_{10}H_{22}$, 98%, Honeywell) as internal standard. D-(+)-glucose (GLU, 99%, Sigma Aldrich), D-(-)-fructose (FRU, 99%, Sigma Aldrich), D-(+)-mannose (MAN, 99%, Sigma Aldrich) and methyl-D-glucopyranoside (MGP, 99%, Sigma Aldrich) were used as standards for HPLC analysis. Pyridine (anhydrous, 99.8%, Sigma-Aldrich) and KBr (Spectroscopy grade, Fisher Chemical) were used in DRIFT spectroscopic studies.

TABLES

Table S1. Stoichiometric coefficients considered in the calculation of product yields –carbon basis– as a function of the starting substrate.

	Hexoses	Pentoses	DHA	Glycolaldehyde
MLA	2	1.66	1	0.66
MG	3	2.5	1.5	1
GADMA	3	2.5	1.5	1
MVG	1.5	1.25	0.75	0.5
MLE	1.2	1	0.6	0.4
MMHB	1.5	1.25	0.75	0.67
MMF	1	1.2	0.5	0.33
HMF	1	1.2	0.5	0.33
DHA	2	1.66	-	0.66
C6-glycosides	1	-	-	-
C5-glycosides	-	1	-	-

Table S2. Composition of hemicellulose hydrolysate recovered from different biomass sources as determined through NREL/TP-510-42623 standard. Extraction and recovery efficiencies for pentoses and hexoses.

Scots Pine			White Birch			Sugarcane Bagasse		
Carbohydrates		Conc. (%)	Carbohydrates		Conc. (%)	Carbohydrates		Conc. (%)
Glucose		15.3	Glucose		5.1	Glucose		9.8
Mannose		30.8	Mannose		2.9	Mannose		0.0
Galactose		9.4	Galactose		5.9	Galactose		3.1
Xylose		19.9	Xylose		69.6	Xylose		68.2
Arabinose		8.1	Arabinose		3.2	Arabinose		8.0
Oligomers C_6		4.2	Oligomers C_6		1.6	Oligomers C_6		0.0
Oligomers C_5		8.1	Oligomers C_5		11.6	Oligomers C_5		15.7
Extraction	C_5	86.5	Extraction	C_5	88.1	Extraction	C_5	93.1
(%)	C_6	88.0	(%)	C_6	87.6	(%)	C_6	76.9
Recovery	C_5	77.1	Yield	C_5	79.5	Yield	C_5	80.1
(%)	C_6	81.6	(%)	C_6	87.5	(%)	C_6	75.5

Different values between extraction and recovery are due to some C5 sugars are extracted but converted into furfural and humins, and some C6 sugars are extracted but converted into HMF, levulinic acid and humins.

Table S3. Impurities and their concentration found in GLV-organosolv Scots Pine hemicellulose hydrolysate.

		Scots Pine
Na	($mg \cdot kg^{-1}$)	7.1
K	($mg \cdot kg^{-1}$)	31.0
Ca	($mg \cdot kg^{-1}$)	139.7
Mg	($mg \cdot kg^{-1}$)	13.2
5-HMF	($g \cdot L^{-1}$)	<0.01
GVL	($g \cdot L^{-1}$)	<0.01

SUPPLEMENTARY SCHEMES AND FIGURES

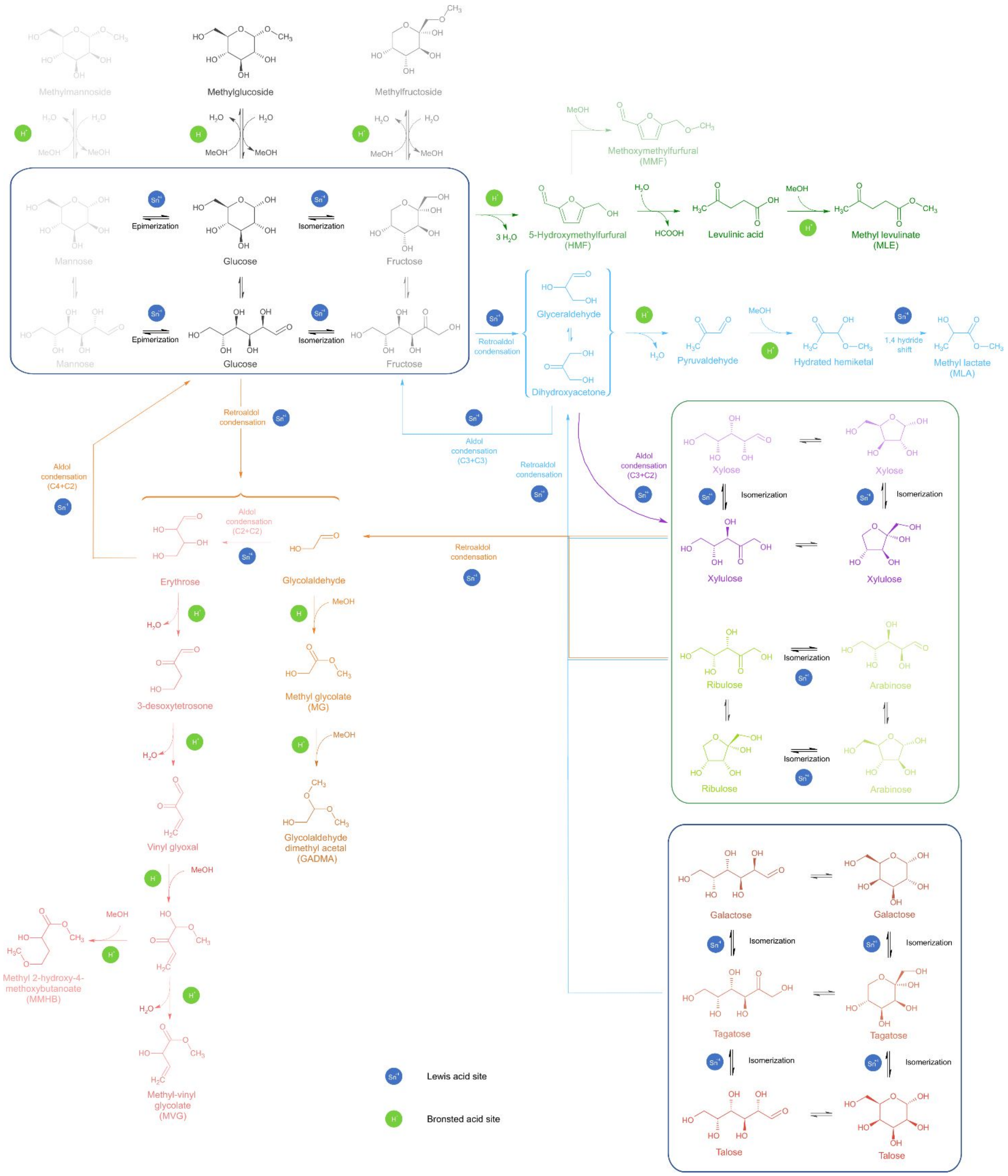

Scheme S1. Reactions pathways taking place in the transformation of hemicellulose monosaccharaides in methanol.

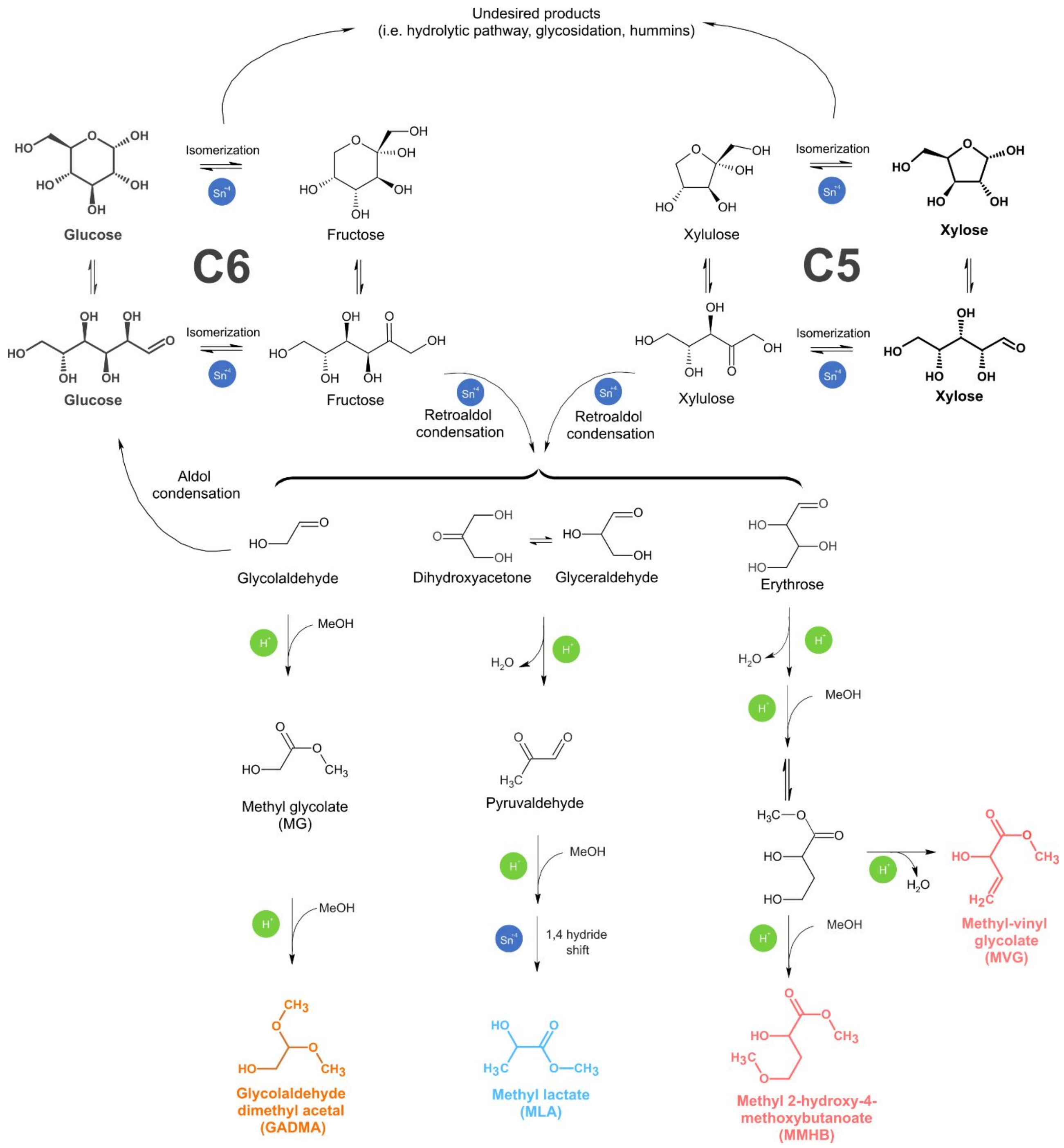

Scheme S2. Simplified scheme highlighting the principal transformations and products in the transformation of hemicellulose monosaccharaides in the presence of [K]Sn-USY and [K]Sn-β zeolites in methanol media.

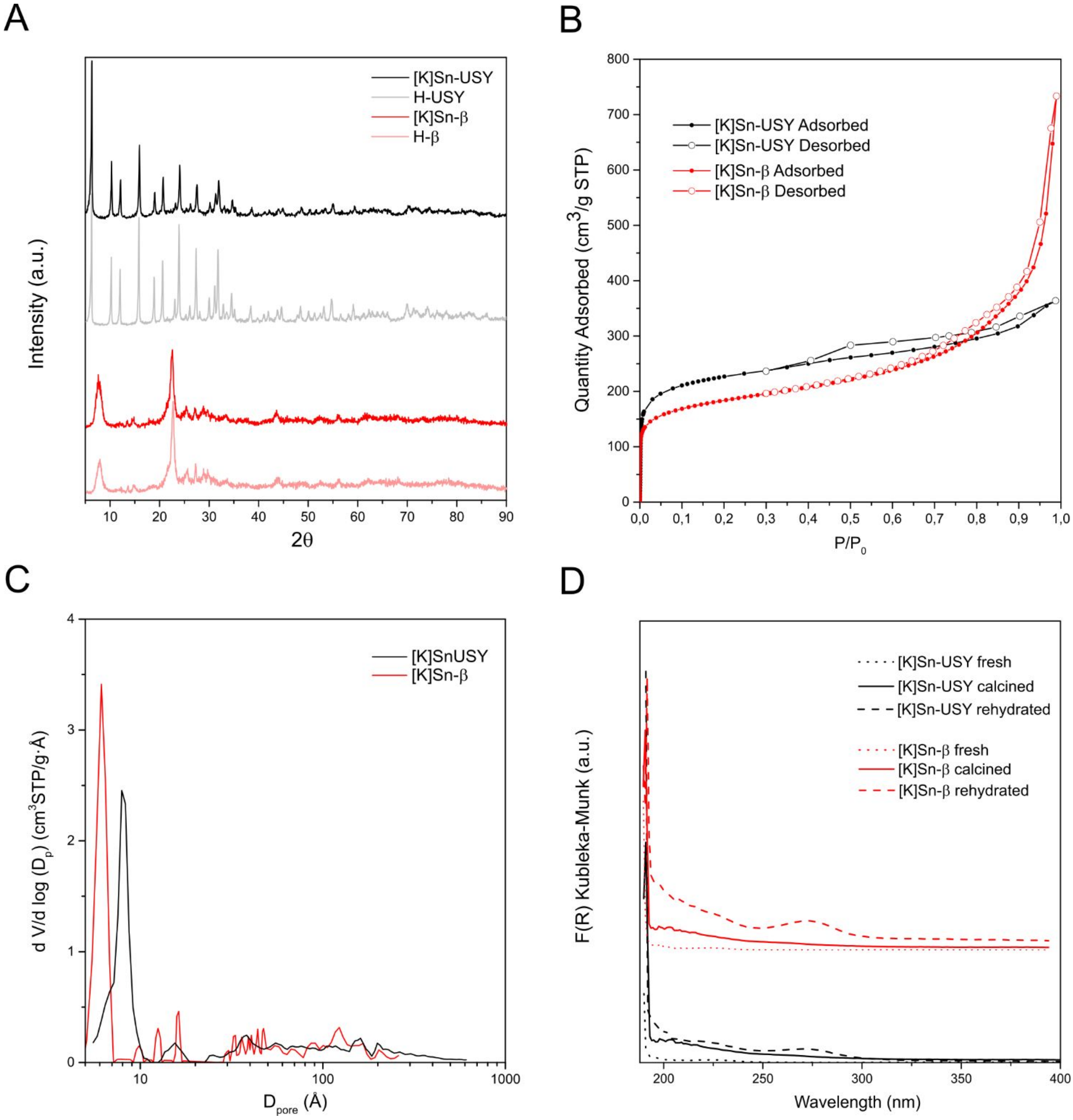

Figure S1. Ar adsorption-desorption isotherms (A), pore size distribution (B) and DR-UV-Vis spectra (C) comparison for both Sn-USY and Sn-β catalyst.

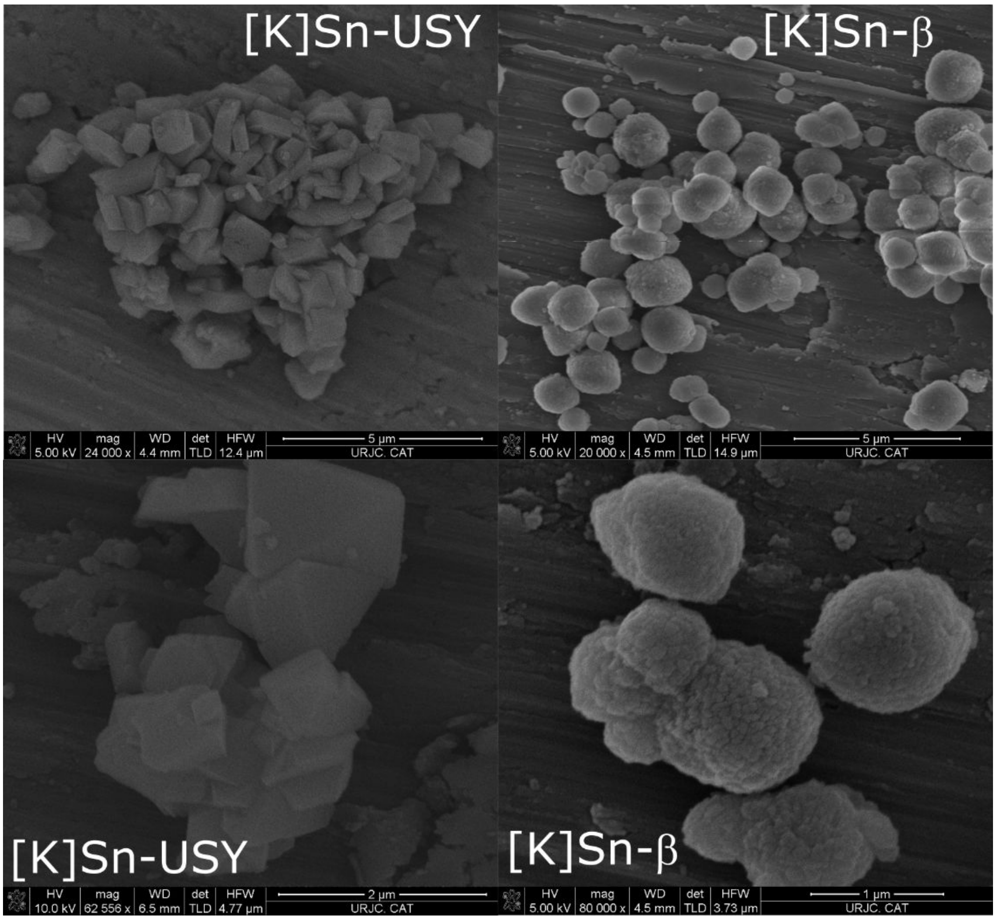

Figure S2. SEM micrographs collected for [K]Sn-USY (left) and [K]Sn-β (right) zeolites at different magnifications.

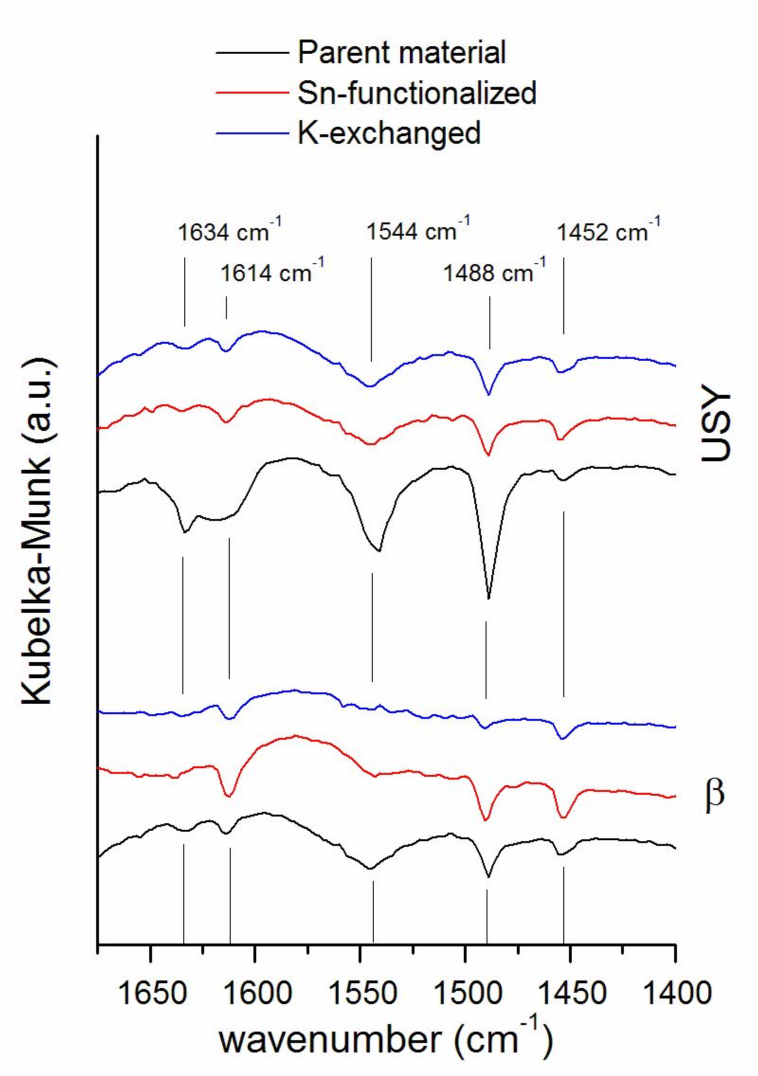

Figure S3. DRIFT spectra of pyridine adsorbed on tested zeolites (parent, Sn-functionalized, and K-exchanged) with faujasite and BEA structures.

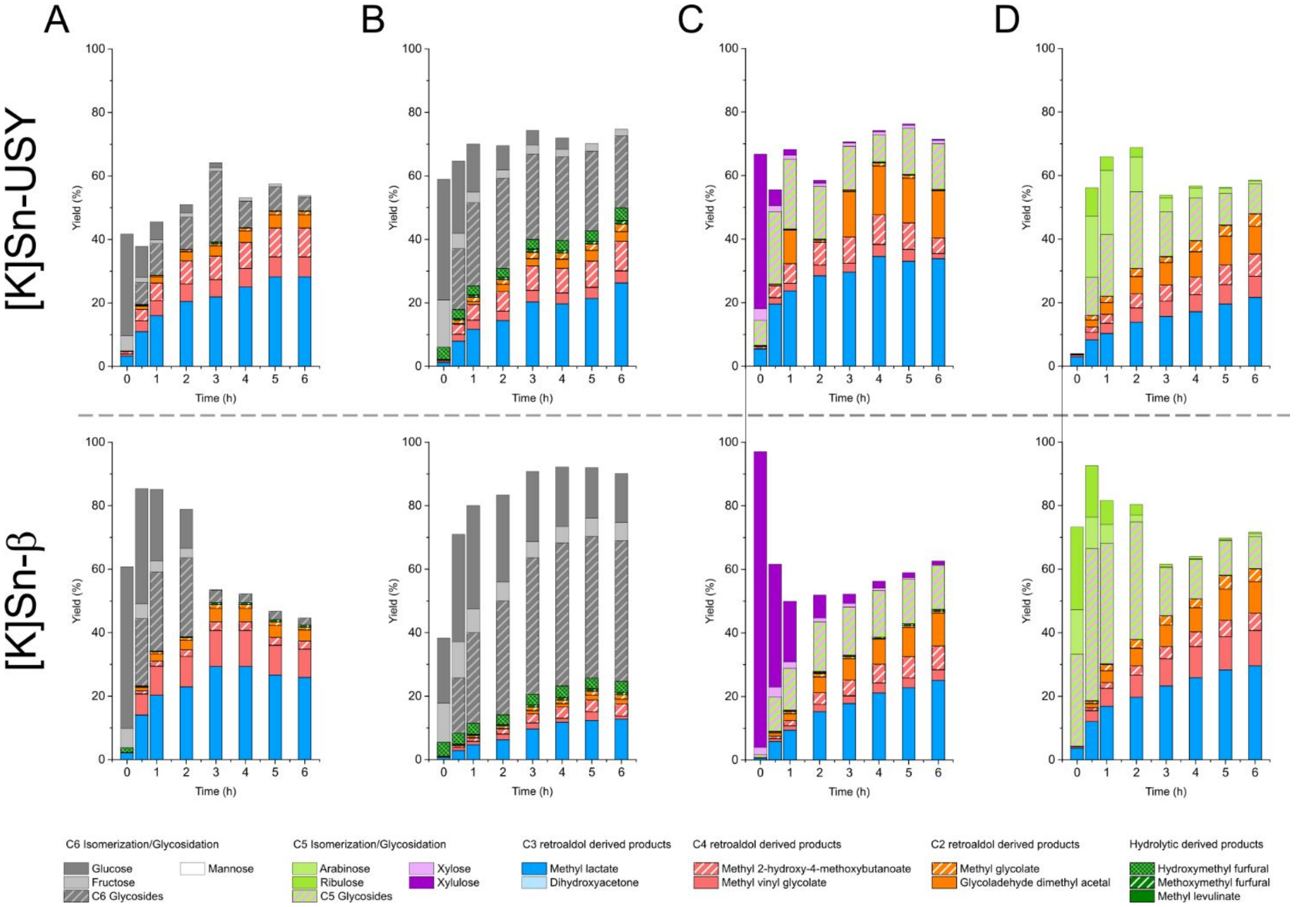

Figure S4. Product distribution obtained in the second use of Sn-USY and Sn-β catalyst in the transformation of hemicellulose monosaccharides (A, glucose; B, mannose; C, xylose and D, arabinose) in methanol. Conditions: monosaccharide concentration = 48 g·L^{-1}; catalyst loading = 0.5g; reaction volume = 50 mL; 150°C; 13 bar (autogenous pressure); 6h reaction time.

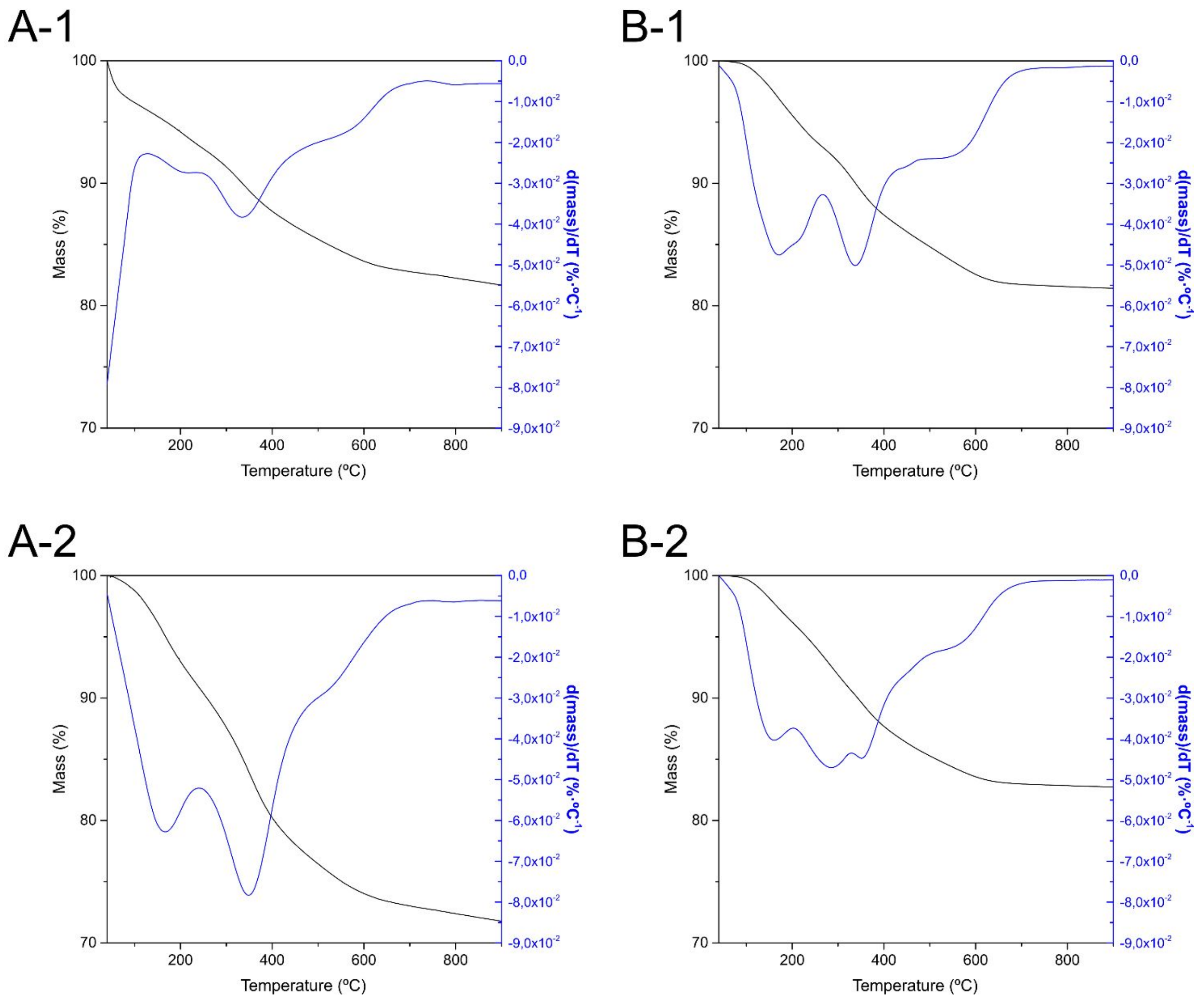

Figure S5. Thermogravimetric analysis of spent catalyst, Sn-USY (1) and Sn-β (2), after catalyst reuse in the transformation of mannose (A) and arabinose (B) in methanol.

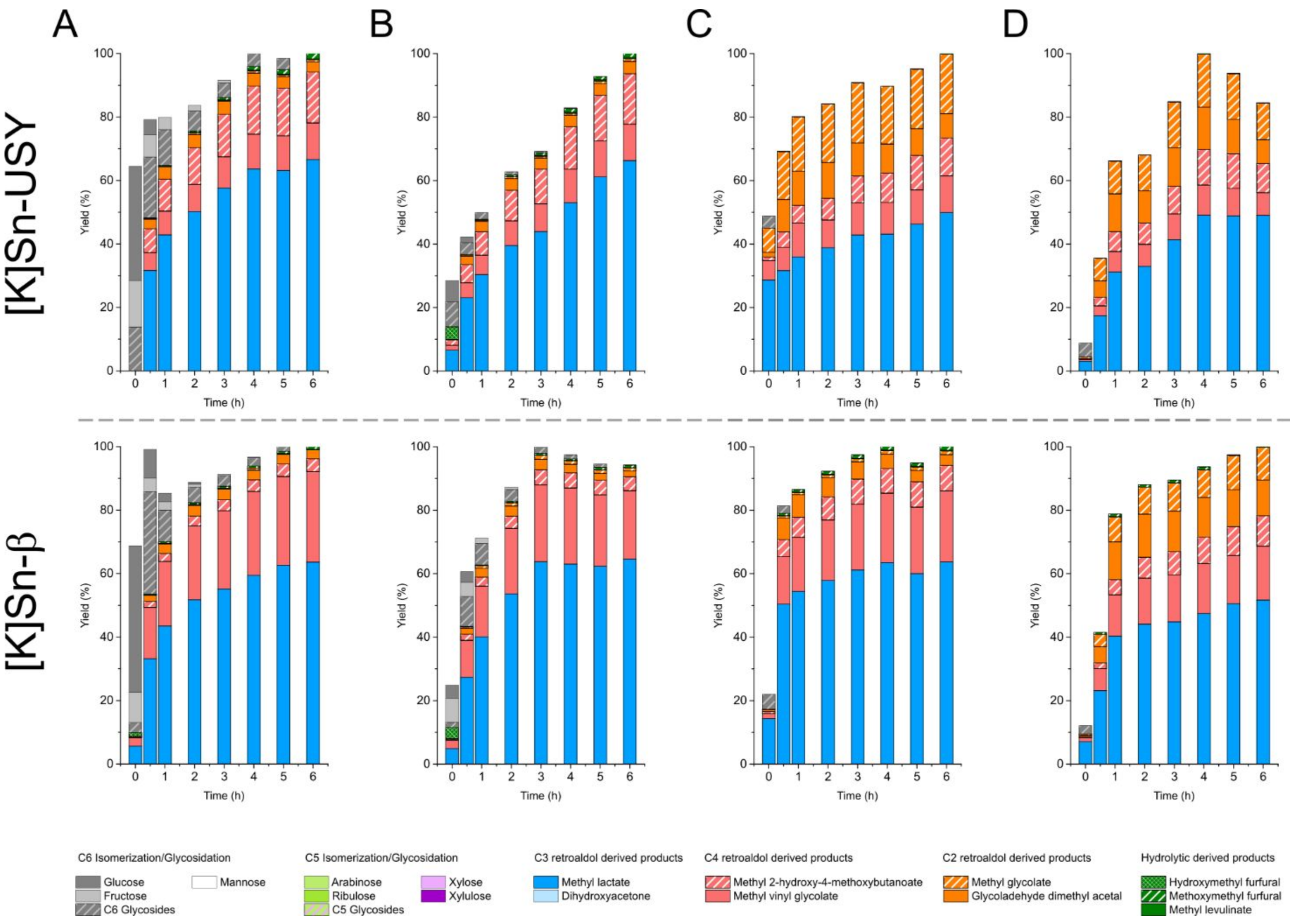

Figure S6. Product distribution obtained in the second use of Sn-USY and Sn-β catalyst in the transformation of hemicellulose monosaccharides (A, glucose; B, mannose; C, xylose and D, arabinose) in methanol:water (96:4wt%) media. Conditions: monosaccharide concentration = 48 $g \cdot L^{-1}$; catalyst loading = 0.5g; reaction volume = 50 mL; 150°C; 13 bar (autogenous pressure); 6h reaction time.

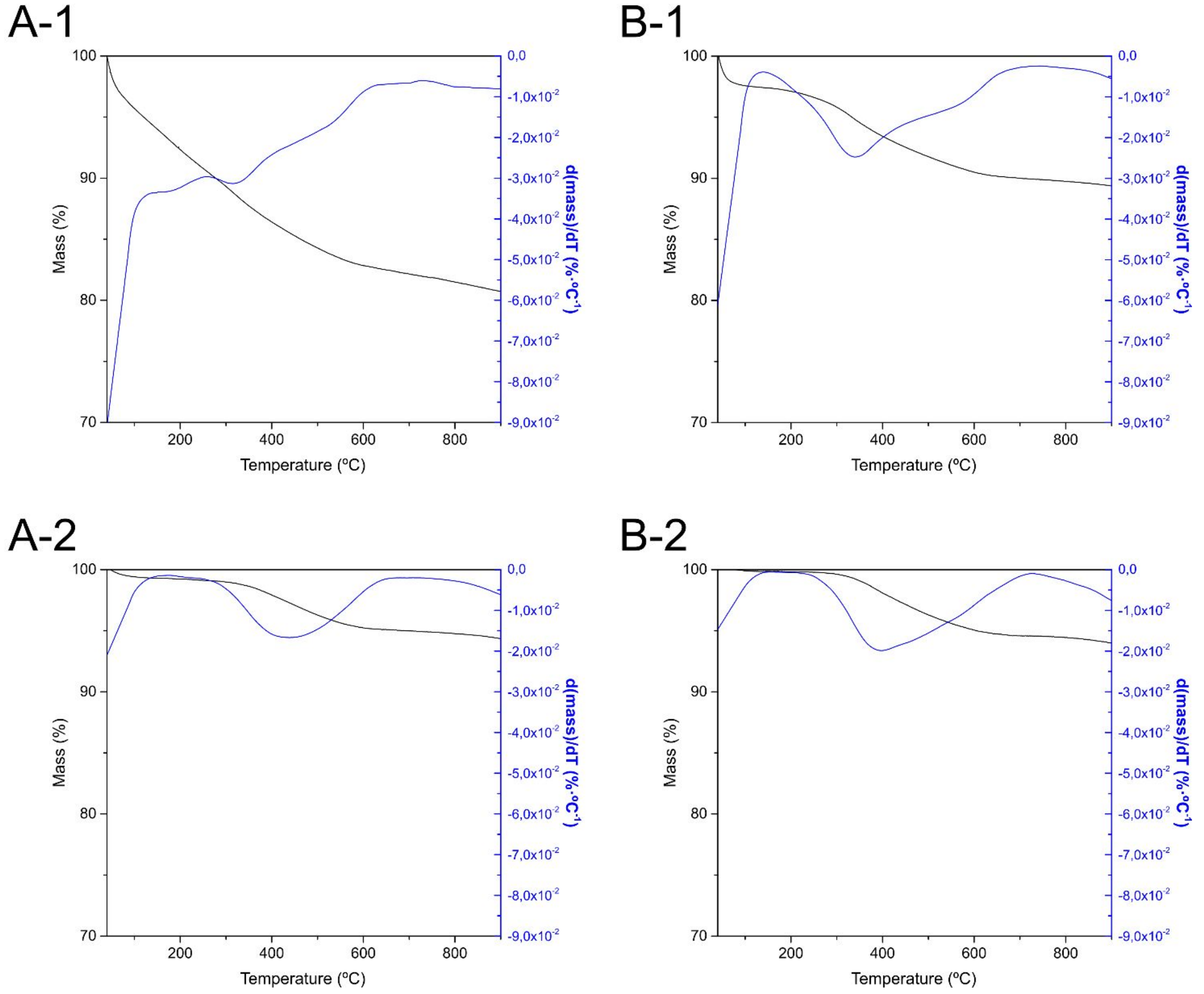

Figure S7. Thermogravimetric analysis of spent catalyst, Sn-USY (1) and Sn-β (2), after catalyst reuse in the transformation of mannose (A) and arabinose (B) in methanol:water (96:4wt%) media.

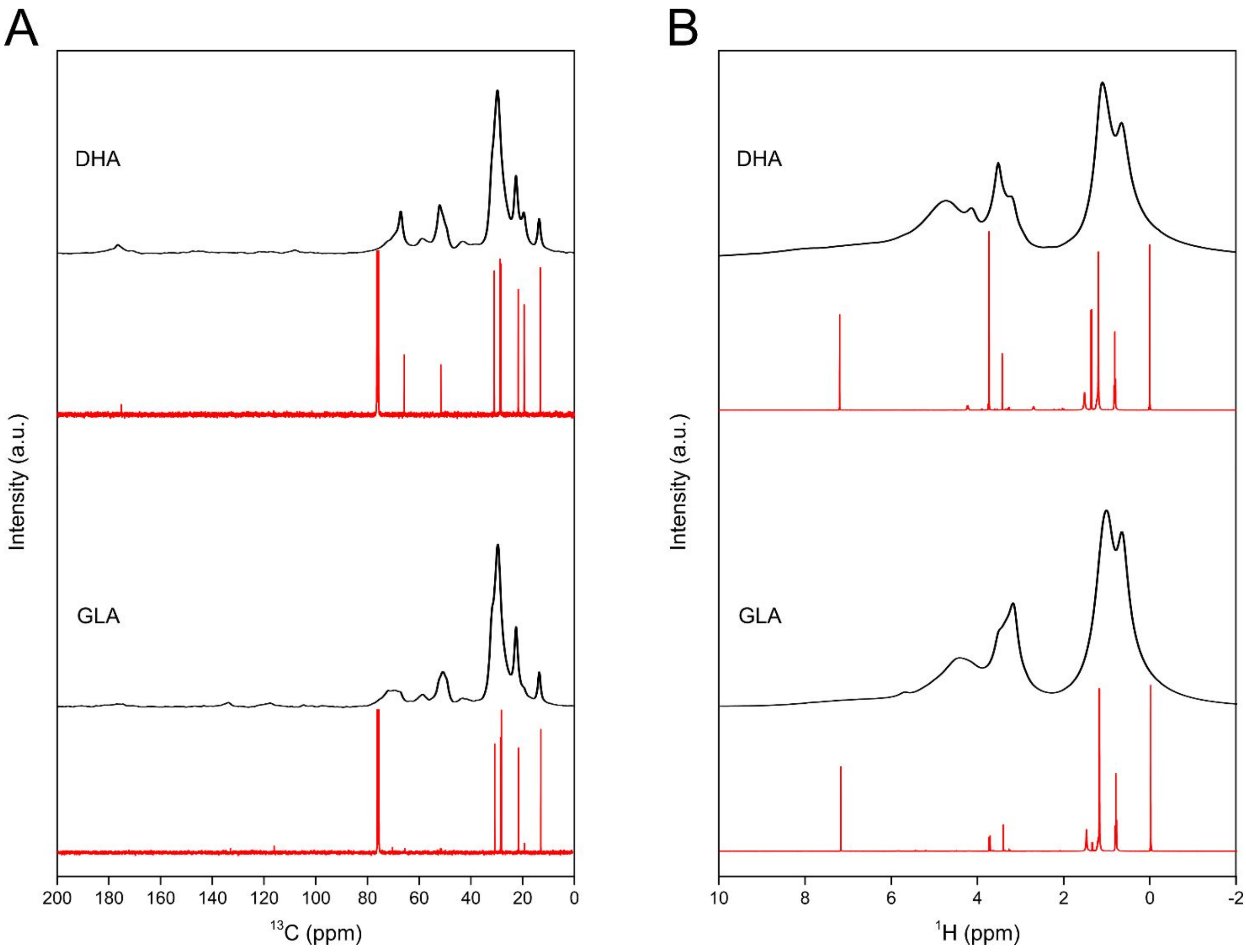

Figure S8. ^{13}C (A) and ^{1}H (B) NMR spectra of Sn-β spent catalyst in the transformation of dihydroxyacetone (DHA) and glycolaldehyde (GLA) (black solid lines) and the liquid-extracted compounds after washing with CD_3Cl (solid red lines)
